# Effect of Physician Gender and Race on Simulated Patients’ Ratings and Confidence in Their Physicians: A Randomized Trial

**DOI:** 10.1001/jamanetworkopen.2019.20511

**Published:** 2020-02-21

**Authors:** Rachel E. Solnick, Kyle Peyton, Gordon Kraft-Todd, Basmah Safdar

**Affiliations:** 1 National Clinical Scholars Program, Institute for Healthcare Policy and Innovation, University of Michigan, Ann Arbor; 2 Department of Emergency Medicine, University of Michigan Medical School, Ann Arbor; 3 Yale Law School, New Haven, Connecticut; 4 Department of Psychology, Boston College, Chestnut Hill, Massachusetts; 5 Department of Emergency Medicine, Yale School of Medicine, New Haven, Connecticut

## Abstract

**Importance:**

Women and black physicians encounter workplace challenges because of their gender and race. It is unclear whether these individuals are assessed with lower patient satisfaction or confidence ratings compared with white male physicians.

**Objective:**

To examine whether physician gender and race affect participant ratings in scenarios in which physician competence is challenged.

**Design, Setting, and Participants:**

This randomized trial enrolled a geographically diverse sample of 3592 online respondents in the United States who were recruited from 2 crowdsourcing platforms: Amazon Mechanical Turk (n = 1741) and Lucid (n = 1851). A 2 × 2 factorial design for the gender and race of simulated physicians was conducted between March 9 and July 25, 2018. Participants were excluded before intervention if they were younger than 18 years, were pregnant, or had a history of cancer or abdominal surgical procedures.

**Interventions:**

A clinical vignette was presented to the participant with a picture of the emergency department physician. Participants were randomly assigned to physicians with different gender and race, with 823 assigned to black women, 791 to black men, 828 to white women, and 835 to white men. A contradictory diagnosis from an online symptom checker introduced doubt about the clinical diagnosis.

**Main Outcomes and Measures:**

A composite outcome (range, 0-100, with 0 representing low patient confidence and satisfaction and 100 representing the maximum on the composite scale) measured participant (1) confidence in the physician, (2) satisfaction with care, (3) likelihood to recommend the physician, (4) trust in the physician’s diagnosis, and (5) likelihood to request additional tests.

**Results:**

Among 3277 adult participants, complete data were available for 3215 (median age, 49 years [range, 18-89 years]; 1667 [52%] female; 2433 [76%] white). No significant differences were observed in participant satisfaction and physician confidence for the white male physician control physicians (mean composite score, 66.13 [95% CI, 64.76-67.51]) compared with white female (mean composite score, 66.50 [95% CI, 65.19-67.82]), black female (mean composite score, 67.36 [95% CI, 66.03-68.69]), and black male (mean composite score, 66.96 [95% CI, 65.55-68.36]) physicians. Machine learning with bayesian additive regression trees revealed no evidence of treatment effect heterogeneity as a function of participants’ race, gender, racial prejudice, or sexism.

**Conclusions and Relevance:**

No significant differences were observed for simulated patients’ evaluations of female or black physicians, suggesting that bias in favor of white male physicians is negligible in survey-based measures of patient satisfaction.

**Trial Registration:**

ClinicalTrials.gov Identifier: NCT04190901

## Key Points

**Question:**

In a simulated clinical encounter, do participants evaluate physicians differently based on the physician’s gender or race?

**Findings:**

In this randomized trial of 3592 online respondents, simulated physician gender and race did not significantly affect participant satisfaction or confidence in physician clinical judgment compared with a white male physician control.

**Meaning:**

Participants reported equal satisfaction and confidence in the simulated physicians’ diagnosis and treatment plans regardless of the physician’s gender or race.

## Teaser

This randomized trial examines whether physician gender and race affect patient ratings of satisfaction and confidence in the physician during a simulated clinical encounter for an emergency department visit.

## Introduction

Women and minority group physicians have steadily become a larger proportion of the health care workforce during the past few decades.^1,2^ However, the same groups report experiencing workplace bias from their institutions, superiors, and colleagues in the form of unfair treatment, leading to unequal compensation and career advancement.^3,4,5,6,7^ They also report discrimination from patients; minority group physicians experience repeated microaggressions and sometimes glaring instances of racism, with patients refusing care, whereas female physicians have reported cases of gender harassment from patients.^8,9,10,11,12^ Such treatment devalues underrepresented physician groups and negatively influences their career trajectories, professional attainment, and retention in medicine.^13,14,15^

Whether physicians’ experience of discrimination from patients represents occasional but offensive anecdotes or signals broader systemic bias that could influence ratings of physicians remains an open question. Evidence from patient-based evaluations is mixed; studies^16,17,18,19,20,21,22^ have variably demonstrated that patients favor male physicians, favor female physicians, or are indifferent. A meta-analysis of 45 studies,^16^ mostly from the primary care setting, that pooled evaluations from more than 100 000 patients for more than 4000 physicians (one-third female) found negligible differences in patient preferences for physician gender. Although some studies^23,24^ have examined the benefits of racial concordance in patient-physician relationships, there is little evidence about the causal effect of a physician’s race or gender on patient-based evaluations.^23,24^ It is therefore unclear whether the discrepancy in patients’ preferences for race-concordant physicians is caused by differences in communication styles or choice of outpatient practice setting where patients have an opportunity to exercise their preferences.

There are also limited data on how patient preferences might influence their evaluations of physicians in emergency departments (EDs), where patients have little choice for physician gender or race.^17,22^ Similarly, communication styles in real-life encounters and extant observational studies make it difficult to isolate the specific causal effect of physician gender and race on patient satisfaction.^20,25,26,27,28^ Herein, we report on 2 randomized experiments that directly examined whether physicians are evaluated differently because of their gender and race using a clinical vignette in which a simulated physician’s competence was challenged by an online symptom checker during an ED visit.

## Methods

### Study Design and Setting

This randomized trial was deemed to be exempt from review by the institutional review board at Yale University, New Haven, Connecticut, because the data were deidentified; written informed consent was obtained from participants before participation. The trial protocol is given in Supplement 1. This trial followed the Consolidated Standards of Reporting Trials (CONSORT) reporting guideline.^29^

We conducted 2 randomized experiments using online ED-based clinical vignettes that independently manipulated physician characteristics in a 2 × 2 factorial design between March 9 and July 25, 2018. An important advantage of an ED-based design is that, unlike in primary care settings, patients in the ED do not have an opportunity to exercise their preference for a particular physician. Previous work has shown that, when given the option, patients are more likely to select a race-concordant physician and satisfaction is higher among patients who had a physician of the same race.^23^ Thus, the physician assignment in the ED might expose biases that would have been filtered out by a patient’s selection of their physician in other settings. Furthermore, a visit to the ED is a higher stress environment than an office setting, and studies have shown that individuals are more likely to make decisions based on racial stereotypes when experiencing a higher cognitive load.^30^

### Participants

We recruited a sample of individuals in the United States (aged ≥18 years) from Amazon Mechanical Turk (MTurk) in March 2018, in which we oversampled older participants (median age, 50 years; range, 19-89 years) using MTurk features to better approximate age groups in the ED.^31^ Although social experiments using MTurk have found similar treatment effects and higher data quality compared with nationally representative samples, the MTurk population tends to be younger, more liberal, and more educated than national samples.^32,33,34,35^ We therefore conducted a direct replication of the first experiment on a more representative group of participants that were quota sampled to match US Census demographics using Lucid, with a participant median age of 45 years (range, 18-86 years) (study 2) in July 2018.^36^

The clinical vignette involved a diagnosis of gastroenteritis based on symptoms as evaluated by an emergency medicine physician. We excluded participants who reported pregnancy, a current or previous diagnosis of cancer, or a history of abdominal surgery. These conditions predispose patients to alternate high-risk diagnoses, which would have made the benign workup given in the vignette unrealistic to a real-world ED evaluation and potentially less credible to participants.^37^ We paid all MTurk participants $1.00 in compensation. Participants in Lucid were paid directly by the vendor either in US dollars or through a points program at a similar rate. Each study took approximately 10 minutes to complete.

### Study Procedures

Participants used their personal computers to access the study administered using the Qualtrics software platform (Qualtrics). Participants gave consent while blinded to the study objectives, and they self-reported data on demographic characteristics, health insurance, trust in physicians, self-assessed health, and frequency of ED visits in a short background survey administered before the clinical vignette. Race/ethnicity was self-reported from options based on the National Institutes of Health reporting guidelines.^38^

**Figure 1.**
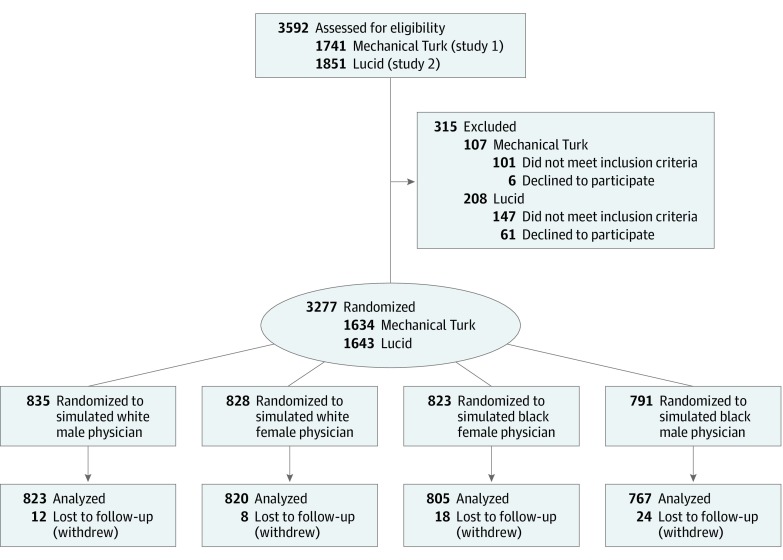
CONSORT Flow Diagram of Participants Through the Trial

**Figure 2.**
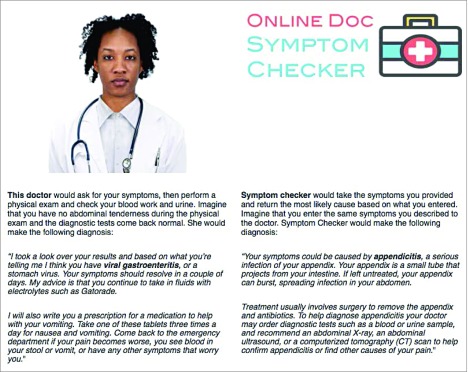
Treatment Vignette Photograph reproduced with permission from the Chicago Face Database.^41^

After completing the background survey, participants were asked to play the role of a patient reporting to the ED with symptoms consistent with gastroenteritis (eFigure 1 in Supplement 2). Accurate comprehension was assessed using recall of case details (eFigure 2 and eFigure 3 in Supplement 2).^39,40^ If participants did not correctly identify case details, they were shown their symptoms a second time to enhance attentiveness and comprehension. Participants were then randomly assigned to 1 of 4 possible treatment arms that would determine the gender and race of the putative ED physician (Figure 1). Participants were then presented with the simulated physician’s image and a written diagnosis of gastroenteritis with a conservative treatment plan, alongside a contradictory diagnosis of possible appendicitis from an Online Doc Symptom Checker (a fabrication created for the purposes of this survey experiment) with a more aggressive treatment plan (Figure 2). Participants then evaluated the putative physician on the primary and secondary outcome measures specified in the preanalysis plans (eMethods 4 and eMethods 7 in Supplement 2). To avoid priming participants to the goals of the study, validated measures of sexism^42,43^ and racial prejudice^44,45^ were asked at the end of the study after primary and secondary outcomes were measured (eMethods 5 and eFigures 12-17 in Supplement 2).

### Physician Image Selection and Randomization

We created a stimulus set of physicians using images from the Chicago Face Database,^46^ a research database for photographs of real human faces of varying gender/ethnicity that have been prerated by independent judges on a variety of dimensions (eg, attractiveness). Stimulus images were selected from the Chicago Face Database to minimize differences in observable traits that might influence ratings of confidence and satisfaction using the following constraints: (1) age between 27 and 39 years (younger physicians are more likely to experience discrimination),^47^ (2) accurate perception of race and gender with at least 90% agreement across prerated judges, and (3) displaying neutral levels of trustworthiness and attractiveness according to prerated judges.

To decrease the likelihood that observed effects would be attributable to other idiosyncratic features of a particular face, we created a set of 10 images for each treatment arm (eg, 10 black men), for a total of 40 images. Simple random assignment was conducted at the participant level using the randomizer tool in Qualtrics. Each participant was first assigned to 1 of 4 possible treatment arms with equal probability: black female (n = 823), black male (n = 791), white female (n = 828), or white male (n = 835). Within each treatment arm, participants saw 1 simulated physician that was randomly selected from the set of 10 images with equal probability using simple random assignment (eMethods 1 and eMethods 2 in Supplement 2).

### Primary Outcome Measures

To provide an overall summary of the effects on participant evaluations of the simulated physicians, we created a composite index (range, 0-100) by extracting the first principal component from a principal component analysis on all 5 preregistered primary outcome measures (patient confidence, patient satisfaction, likelihood to recommend, believes symptom checker, and requests more tests). Reporting a composite score for patient experience facilitates interpretability and is a method used by the Consumer Assessment of Health Plans Study survey for items, with Cronbach α coefficients greater than 0.70 indicative of good reliability.^48^ All 5 primary outcome measures were highly correlated in both study 1 (α = 0.81) and study 2 (α = 0.73).

*Patient confidence* is the unweighted mean of participants’ responses to 2 questions (from 1 [lowest] to 5 [highest] confidence): (1) “How confident are you that this physician made the correct diagnosis?” and (2) “How confident are you that this physician recommended the correct treatment plan?” *Patient satisfaction* is a single item scaled from 1 (lowest) to 10 (highest): “What number would you use to rate your care during this emergency room visit?” *Likelihood to recommend* is a single item scaled from 1 (definitely not) to 5 (definitely): “Would you recommend this physician to your friends and family?” *Believes symptom checker* is a binary response (0 [the symptom checker], 1 [the physician]) to the question: “Which diagnosis do you think is more likely to be correct?” *Requests more tests* is a single item scaled from 1 (definitely) to 5 (definitely not): “Would you ask the doctor to perform additional diagnostic tests?”

Patient confidence, believes symptom checker, and requests more tests were designed to capture the patients’ confidence and willingness to challenge the physician’s expertise when presented with contradictory information by an outside source of medical advice (ie, an online symptom checker). Patient satisfaction and likelihood to recommend are global ratings of satisfaction from the Hospital Consumer Assessment of Healthcare Providers and Systems (HCAHPS) and the Press Ganey survey, the most commonly used surveys of patient experience.^49,50^ eMethods 4 in Supplement 2 gives complete details on all survey items used to construct the primary outcome measures, with screenshots of how they appeared to participants in the vignettes (eFigures 4-7 in Supplement 2) .

### Secondary Outcome Measures

Studies 1 and 2 also included preregistered secondary outcome measures of perceived warmth and competence of the simulated physicians (eFigure 8 and eFigure 9 in Supplement 2). These measures have been used to study patient-physician relationships and capture 2 dimensions of stereotype content in social perception.^51,52^ Study 1 also included a measure of the perceived fairness of a $350 charge for the ED visit (eFigure 10 in Supplement 2), and study 2 included measures for willingness to complain and sue the physician if a diagnostic error resulted in an adverse outcome (eFigure 11 in Supplement 2) because previous studies have identified increased medicolegal action against physicians who belong to minority groups.^53,54^

### Statistical Analysis

We estimated treatment effects using ordinary least-squares regression on the 4-level treatment factor, with white male as the omitted reference category. To maximize the precision of estimated treatment effects, we used covariate-adjusted ordinary least squares on the stacked data set of both experiments.^55^ Covariates used in adjusted regression were measured pretreatment and included participant-level demographic characteristics (age, race/ethnicity, gender, and college education), self-reported trust in physicians, mental and overall health, insurance status, unpaid medical bills, and frequency of ED visits in the previous 6 months. We also added a study fixed effect (binary indicator) to adjust for differences across studies. We reported estimated treatment effects for the composite index and the 5 underlying primary outcome measures.

To facilitate interpretation, estimates were standardized using a Glass delta, which scaled outcomes by the SD in the white male control group. Results are presented graphically with 90% CIs and 95% CIs and a margin of equivalence bound within 0.20 standard units, which corresponds to an effect size of approximately one-fifth of 1 SD. The null hypothesis of nonequivalence is rejected in favor of equivalence when a 90% CI is contained within the margin of equivalence, and the null hypothesis of no significant difference from 0 is rejected if a 95% CI excludes 0. With 700 participants per treatment arm (N = 2800), the minimum detectable effect at 80% power using a 2-sided hypothesis test (at α = .05) is approximately 0.15 standardized units for any between-group difference. Combining the results from these 2 testing procedures assisted us in ruling out the presence of effects larger than the margin of equivalence, which was the smallest effect size of interest in this study.^56^ We concluded that an estimated effect was negligible (bounded between –0.20 and 0.20 standard units) when the 90% CI was inside the margin of equivalence and the 95% CI included 0.

We also examined whether certain subgroups of participants may have been affected differently by treatment using bayesian additive regression trees (BARTs), a machine learning algorithm that estimates treatment effect heterogeneity as a function of each participant’s covariate profile by including multiple potential moderators in the same model.^57,58^ We preregistered this BART analysis for demographic covariates as well as measures of racial prejudice and sexism. To provide an overall summary of treatment effect heterogeneity, we plotted BART-estimated treatment effects with 95% credible intervals for each individual. Intervals that excluded 0 provided evidence in support of treatment effect heterogeneity. R version 3.5.1 (R Project for Statistical Computing) statistical software was used for statistical analyses, and the dbarts package was used for BARTs. eMethods 6 in Supplement 2 provides additional details on BART implementation.

## Results

Of the 3277 randomized participants, 3215 (representing all contiguous US states) completed the survey (Figure 1). In this combined sample, participants’ median age was 49 years (range, 18-89 years), 52% (1667 of 3215) were female, 76% (2433 of 3215) were white, and 10% (333 of 3215) were black. The Table reports background characteristics for study 1 (MTurk), study 2 (Lucid), and the pooled sample. Approximately 40% of participants in study 1 and 34% in study 2 endorsed some group-level superiority of white individuals compared with the black individuals (eMethods 5 in Supplement 2). We did not find evidence that loss to follow-up was associated with imbalance in background characteristics by treatment arm for either study (eMethods 3 and eTables 1-3 in Supplement 2).

**Table.** Baseline Characteristics of Study Participants^a^

Characteristic	Combined (N = 3215)	Study 1 (n = 1619)	Study 2 (n = 1596)
Age, median (range), y	49 (18-89)	50 (19-89)	45 (18-86)
Female	1667 (51.85)	873 (53.92)	794 (49.75)
College educated	1515 (47.12)	818 (50.53)	697 (43.67)
Household income below median level	2086 (66.10)	967 (59.95)	1119 (72.52)
Race/ethnicity			
White non-Hispanic	2433 (75.68)	1300 (80.30)	1133 (70.99)
Black	333 (10.36)	157 (9.70)	176 (11.03)
Hispanic	206 (6.41)	64 (3.95)	142 (8.90)
Other	243 (7.56)	98 (6.05)	145 (9.09)
Insurance			
Medicaid	404 (12.57)	34 (2.10)	370 (23.18)
Medicare	362 (11.26)	121 (7.47)	241 (15.10)
Uninsured	420 (13.06)	229 (14.14)	191 (11.97)
Unpaid medical bills	735 (22.86)	384 (23.72)	351 (21.99)
≥1 Emergency department visit in past 6 mo	609 (18.94)	240 (14.82)	369 (23.12)
Mental health, mean (SD)^b^	3.60 (1.12)	3.65 (1.11)	3.54 (1.12)
Overall health, mean (SD)^c^	3.43 (0.96)	3.44 (0.95)	3.41 (0.98)
Trust in physicians, mean (SD)^d^	3.88 (0.81)	3.89 (0.87)	3.88 (0.75)

^a^ Data are presented as number (percentage) of participants unless otherwise indicated.

^b^ Mean score on validated Likert score (excellent [5], very good [4], good [3], fair [2], poor [1]) for “In general, how would you rate your mental health?”

^c^ Mean score on validated Likert score (excellent [5], very good [4], good [3], fair [2], poor [1]) for “In general, how would you rate your overall health?”

^d^ Mean score on validated Likert score (strongly agree [7], agree [6], somewhat agree [5], neither agree nor disagree [4], somewhat disagree [3], disagree [2], strongly disagree [1]) for “How much do you agree or disagree with the following statement: All things considered, doctors in the United States can generally be trusted.”

### Primary Outcomes

**Figure 3.**
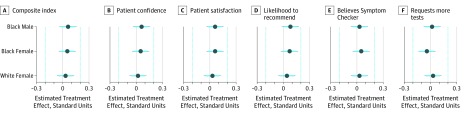
Estimated Treatment Effects of Race and Gender of Simulated Physicians on Composite Index and Primary Outcome Measures Covariate-adjusted treatment effects from ordinary least squares regression with control group (simulated white male physician). Estimates are pooled across 2 independent patient analog experiments (N = 3215) and standardized using the Glass delta, which scales outcomes by the SD in the control group. Composite index (range, 0-100) was created by extracting the first principal component from a principal component analysis on all primary outcome measures (patient confidence, patient satisfaction, likelihood to recommend, believes symptom checker, and requests more tests) (eMethods 4 and eTable 5 in Supplement 2). Dots indicate means; cyan lines, 90% CIs; gray lines, 95% CIs; and cyan dotted vertical lines, margin of equivalence within 0.20 standard units.

In the combined sample (n = 3215), the unadjusted mean composite index was not statistically distinguishable for any pairwise comparison across treatment arms (white male, 66.13 [95% CI, 64.76-67.51]; black male, 66.96 [95% CI, 65.55-68.36]; black female, 67.36 [95% CI, 66.03-68.69]; white female, 66.50 [95% CI, 65.19-67.82]) (eTable 4 in Supplement 2). Estimated covariate-adjusted treatment effects (estimated against the white male control) (eTable 5 in Supplement 2) on the composite index were also not statistically distinguishable from 0 (white female, 0.03 [95% CI, –0.07 to 0.13]; black female, 0.05 [95% CI, –0.05 to 0.15]; black male, 0.06 [95 % CI, –0.04 to 0.16]). On the basis of the combined results from equivalence tests and null hypothesis tests, we found no detectable effects of physician gender and race and ruled out effects larger than within 0.20 standard units on the composite index and all underlying primary outcome measures (Figure 3). No significant differences were observed when study 1 and study 2 were instead analyzed separately (eTable 6 and eTable 7 in Supplement 2).

### Treatment Effect Heterogeneity

**Figure 4.**
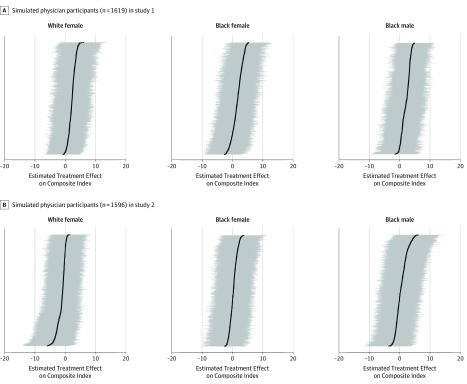
Bayesian Additive Regression Tree (BART)–Estimated Treatment Effects of Race and Gender of Simulated Physicians on Composite Index of Primary Outcomes by Study Black dots indicate the BART-estimated treatment effect for each individual as a function of their covariate profile, ordered by effect size. Grey horizontal lines indicate 95% credible intervals; intervals that exclude 0 would provide evidence of treatment effect heterogeneity. Composite index (range, 0-100) was created by extracting the first principal component from a principal component analysis on all primary outcome measures (patient confidence, patient satisfaction, likelihood to recommend, believes symptom checker, and requests more tests) (eMethods 4 and eMethods 6 in Supplement 2).

Figure 4 plots BART-estimated treatment effects on the composite index for each participant as a function of their individual covariate profile for both MTurk (study 1) and Lucid (study 2) samples. The BART-estimated treatment effects were consistently indistinguishable from 0 and similar across participant samples. This analysis revealed little evidence of variation in BART-estimated treatment effects as a function of participant-level characteristics (Figure 4). The corresponding 95% credible interval did not exclude 0 in any of the cases in which a participant was estimated to have a positive (or negative) treatment effect (eTable 8 in Supplement 2). We therefore did not find compelling evidence that some subgroups of participants (eg, prejudiced white men without a college education who were aged ≥65 years) responded differently to the race and gender of simulated physicians than others.

### Secondary Outcomes

Perceived warmth and competence scales were created in study 1 (warmth: Cronbach α = 0.88; competence: Cronbach α = 0.88) and study 2 (warmth: Cronbach α = 0.89; competence: Cronbach α = 0.94). We did not find evidence of participant bias against black or female physicians on the secondary outcomes of perceived warmth and competence, perceived fairness of ED visit charge, or willingness to sue or complain because of misdiagnosis that resulted in a bad outcome (eMethods 7, eTable 9, and eTable 10 in Supplement 2).

## Discussion

To our knowledge, these were the first large-scale, preregistered, randomized experiments to directly estimate the effect of physician gender and race on simulated patient evaluations in a diverse national sample of US participants. We found that black or female physicians were not rated lower than white male physicians in measures of simulated patient satisfaction or confidence in an ED setting. These findings suggest that survey-based measures of simulated patient confidence and satisfaction may not be systematically negatively affected by physician race and gender.

Of importance, the results reported here should not be interpreted as contradicting the lived experiences of discrimination reported by physicians from underrepresented groups. The absence of a systematic preference for white male physicians in the controlled setting does not diminish the damaging and lasting effect that even a single instance of discrimination from patients or colleagues can have on minority and female physicians.^10,12^ However, we did not find compelling evidence that participants were biased against black or female physicians in the simulated interactions in our study.

The experimental designs we used address several important methodologic challenges present in previous research, including a large sample size, random assignment of physician race and gender, replication across 2 independent studies, and preregistration of primary and secondary outcomes and the statistical analyses. Unlike previous experiments that recruited smaller numbers of undergraduate or medical students as patient analogs, we recruited a large, geographically diverse pool of participants who more closely approximates typical ED patients.^31^ In addition, our stimuli used multiple physician images drawn from a validated stimulus set to minimize differences in observable characteristics (eg, physician attractiveness) that may affect ratings of confidence and satisfaction independent of physician race and gender. Furthermore, we controlled for variability in the clinical content of the encounter by holding constant the simulated physicians’ diagnoses and treatment plans as well as communication styles across treatment arms.

Our results add to a growing body of observational studies investigating patient bias against female and minority group physicians, most of which have not found evidence of systematic bias. For example, an analysis of more than 9000 Press Ganey surveys found no differences in patient satisfaction by ED physicians’ gender.^59^ A meta-analysis focused on observational studies in primary care settings found negligible evidence that patients favored female physicians, an effect attributed to publication bias and gender-specific differences in physician communication styles and patient-centered care.^16^ Many observational studies have examined the association between patient-physician gender-concordance and patient satisfaction, but the results have been mixed across a variety of clinical settings, with some reporting gender concordance preferences and others finding evidence of gender discordance.^47,60,61,62^

Few studies have examined the association of physician race with overall patient evaluations, and the results have been inconsistent depending on the study design. An analysis of more than 51 000 Press Ganey and HCAHPs surveys^63^ from hospital-discharged patients found no difference in patient satisfaction by physician race. In contrast, other observational surveys^23,24^ have shown an association between race concordance and patients’ satisfaction or feeling that a visit was participatory, such that minority group physicians were favored by their minority group patients compared with white physicians. Although minority group physicians may be preferred by minority group patients, a study found that they may experience bias by simulated white patients. In a simulation experiment, participants were shown a physician profile with a randomized name to represent a different race or gender of the physician. White participants were less likely to select a black or middle-eastern physician even though they had the same quality scores compared with the white physician counterpart.^64^

To our knowledge, the only randomized experiment on patient-physician race concordance, conducted in Oakland, California, found that black men assigned to black male physicians took more preventive health measures than black male patients assigned to white male physicians.^65^ Although our experiments were not designed to detect race concordance effects (black participants were 10% of our sample), the BART analysis did not reveal compelling evidence of treatment effect heterogeneity as a function of participants’ background characteristics. This finding suggests that increasing the diversity of the physician workforce is unlikely to decrease patient satisfaction and may improve quality of care for patients from underrepresented minority groups. Adequately powered experimental designs that study the effects of race and gender concordance on quality of care and health outcomes is an avenue for future research.

The importance of creating inclusive and diverse workplaces in health care cannot be overstated because more diverse teams are associated with better patient care, lower mortality, better science, and more successful organizations with higher productivity, innovation, and employee retention.^66,67,68,69^ Thus, there is a renewed call to improve the status quo through institutional-level change to elevate underrepresented groups using accountability measures through organizations such as Time’s Up Healthcare and Men Advocating Real Change.^70,71^ Our study further supports these efforts by suggesting that patient bias against physicians may be less of a driver of workplace discrimination than these other sources.

### Limitations

This study has limitations. First, the experimental designs used written clinical vignettes in a hypothetical ED interaction in which neither the physician nor the patient were real. Simulated encounters cannot capture important characteristics of real-world interactions that might shape patient-physician interactions, such as nonverbal communication and communication style. However, the use of lay participants to play the role of a patient analog is supported by a meta-analysis of communication studies that showed a large overlap between patient analogs and patient perceptions of a clinical encounter.^72^ Furthermore, case vignettes using written descriptions show that physicians make similar assessments from vignettes as they do in real clinical encounters.^73^ The benefit of simulation designs is that they control for complexities introduced by real-world interactions, such as practice styles, which have been found to be independently associated with patient ratings.^25,27^

Furthermore, the use of an ED setting may limit the generalizability of the findings reported here to other clinical contexts. Unlike other contexts, the ED is a unique environment where patient-physician relationships are brief and episodic, and physicians cannot be chosen by patients in advance. Investigating the role that the length of the patient-physician relationship and patient choice play in determining patient satisfaction is an important area for future research.

Moreover, we chose a low-acuity clinical vignette. It is possible that a high-stake encounter could have elicited a different response. However, in our second experiment (study 2), we extended the vignette and the participant was told they underwent emergency surgery because of a misdiagnosis by the physician and had to stay in the intensive care unit. We did not find evidence of race or gender biases in the extent to which patients desired retribution in terms of willingness to sue or complain for the physician’s error. Still, conducting similar studies of situations in which patients experience greater stress or cognitive load is another important avenue for future research.

In addition, it is theoretically possible that some participants may have discerned the purpose of the study and censored their prejudice against female and black physicians to appear more socially desirable, thereby attenuating estimated treatment effects. However, all participants were blinded to the study objectives, and to our knowledge, there is no empirical support for such threats to inference in randomized survey experiments conducted in the anonymous online environment.^74,75^ In addition, our respondents willingly disclosed racial and/or gender prejudice on related measures; for example, approximately 40% of participants in study 1 and 34% of participants in study 2 endorsed some group-level superiority of white individuals vs black individuals (eMethods 5 in the Supplement 2). We did not find evidence that these characteristics were predictive of heterogeneous effects in the BART analysis.

## Conclusions

Using large, survey-based experiments of a simulated ED encounter, we found no detectable effects of physicians’ race or gender on simulated patients’ confidence and satisfaction. These results suggest that institutional biases and workplace dynamics, including potential discrimination from leadership, peers, and staff, may play a greater role in the bias experienced by women and minority group physicians in the ED than clinical encounters with patients.

## Notes

Supplement 1. **Trial Protocol**

Supplement 2. **eMethods 1.** Randomization and Estimation Procedures **eMethods 2.** Additional Design Details **eMethods 3.** Covariate Balance **eMethods 4.** Primary Outcomes **eMethods 5.** Survey Measures of Racial Prejudice and Sexism **eMethods 6.** BART Estimated Treatment Effects **eMethods 7.** Secondary Outcomes **eFigure 1.** Scenario Instructions 1 of 2 **eFigure 2.** Attention Check Drag and Drop (With Correct Responses Displayed) **eFigure 3.** Scenario Instructions 2 of 2 **eFigure 4.** Patient Confidence Measure in Study 1 **eFigure 5.** Patient Satisfaction Measure in Study 1 **eFigure 6.** Patient Confidence Measure in Study 2 **eFigure 7.** Patient Satisfaction Measure in Study 2 **eFigure 8.** Warmth and Competence in Study 1 **eFigure 9.** Warmth and Competence in Study 2 **eFigure 10.** Fairness of Visit in Study 1 **eFigure 11.** Willingness to Publish Doctor Error in Study 2 **eFigure 12.** Example of Explicit Prejudice Survey Item Used in Qualtrics **eFigure 13.** Distribution of Scores on Prejudice Items in Study 1 **eFigure 14.** Distribution of Scores on Prejudice Items in Study 2 **eFigure 15.** Distribution of Scores on Sexism Items in Study 1 **eFigure 16.** Distribution of Scores on Hostile Sexism Items in Study 2 **eFigure 17.** Distribution of Scores on Benevolent Sexism Items in Study 2 **eTable 1.** Background Characteristics by Treatment Group in Study 1 **eTable 2.** Background Characteristics by Treatment Group in Study 2 **eTable 3.** Randomization Inference (RI) for Covariate Balance **eTable 4.** Average Patient Evaluation Scores on Composite Index of Primary Outcomes **eTable 5.** Estimated Treatment Effects on Primary Outcomes in Combined Sample **eTable 6.** Estimated Treatment Effects on Primary Outcomes in Study 1 **eTable 7.** Estimated Treatment Effects on Primary Outcomes in Study 2 **eTable 8.** Summary Statistics for BART-Estimated Treatment Effects on Composite Index (0-100) **eTable 9.** Estimated Treatment Effects on Secondary Outcomes in Study 1 **eTable 10.** Estimated Treatment Effects on Secondary Outcomes in Study 2 **eReferences.**

Supplement 3. **Data Sharing Statement**
