# Supplementary material for: Effect of Physician Gender and Race on Simulated Patients’ Ratings and Confidence in Their Physicians: A Randomized Trial
Source: JAMA Netw Open. 2020 Feb 21;3(2):e1920511. doi: 10.1001/jamanetworkopen.2019.20511 (PMC7043197; doi:10.1001/jamanetworkopen.2019.20511)
Supplement: Supplement 1. — Trial Protocol [file jamanetwopen-3-e1920511-s001.pdf]

Brief Summary:

The purpose of the study was to determine whether the race and gender of a simulated doctor affected analog patients' reported confidence and satisfaction in the simulated doctor's diagnosis and treatment plan. The study used two randomized patient analog experiments.

This study is complete and pre-analysis plans (PAPs) for each experiment were published prior to data collection. The PAPs are available at: http://aspredicted.org/blind.php?x=43xj25 (Study 1) and https://aspredicted.org/blind.php?x=369st7 (Study 2).

Condition or disease	Intervention/treatment
Bias, Racial<br>Bias, Sex<br>Patient Satisfaction<br>Physician-Patient Relations	Behavioral: Simulated Black Male Physician<br>Behavioral: Simulated Black Female Physician<br>Behavioral: Simulated White Male Physician<br>Behavioral: Simulated White Female Physician

Detailed Description:

Prior literature is unclear on whether patients display bias in their evaluations of physicians based on their race or gender. We estimated the effects of physician race and gender using an online clinical vignette. Participants played the role of analog patients reporting to the Emergency Department (ED) with symptoms consistent with gastroenteritis. Participants were provided with a diagnosis of gastroenteritis by a simulated ED physician. The race (black or white) and gender (male or female) of the simulated physician was randomly assigned in a 2x2 factorial experiment. Simulated physicians provided a diagnosis of gastroenteritis and contradicted by an Online Symptom Checker. Following the physician's diagnosis and contradiction by the Online Symptom Checker, participants rated the simulated physician on survey measures of satisfaction and confidence in both the treatment plan and diagnosis. The main (null) hypothesis tested was that there were no differences across the four treatment arms (Black Female, Black Male, White Female, White Male). Participants for the first experiment (Study 1) were

recruited from Amazon Mechanical Turk (MTurk) and participants for the second experiment (Study 2) were recruited from Lucid.

Primary Aim: To determine whether the race and gender of a simulated physician had a causal effect on participants' confidence and satisfaction in the physician's diagnosis and treatment plan in an ED setting.

Exploratory Aims: To determine whether the race and gender of a simulated physician had a casual effect on participants' perceptions of the warmth and competence of the physician, their willingness to sue or complain about the physician for an incorrect diagnosis, and their perceived fairness of the charge for the visit.

Study Design

Study Type:	Interventional
Actual Enrollment:	3592 participants
Allocation:	Randomized
Intervention Model:	Factorial Assignment<br><br>This is a 2x2 experimental design. Subjects were randomly assigned to one of four possible conditions: Black Female, Black Male, White Female, White Male. Within each condition, subjects were randomly assigned 1 of 10 possible putative doctors from a total of 40 putative doctors, 10 for each condition. For example, 10 Black Female doctors, etc. The images of putative physicians were selected from actors in the Chicago Face Database and altered to wear a white coat. Given that the vast majority of Emergency Physicians in the United States are white men, the White Males condition served as the "control".
Masking:	None (Open Label)
Primary Purpose:	Health Services Research
Official Title:	Effect of Physician Race and Gender on Simulated Patients' Ratings and Confidence in Their Physicians: A Randomized Trial
Actual Study Start Date:	March 9, 2018
Actual Primary Completion Date:	July 31, 2018

Actual Study Completion Date: July 31, 2018

Arms and Interventions

Arm	Intervention/treatment
Experimental: Simulated Black Male Physician<br><br>Participants are randomized to view the clinical vignette with a simulated Black Male physician.	Behavioral: Simulated Black Male Physician<br><br>Participants in this arm of the experiment viewed one of 10 randomly selected possible images of a simulated Black Male physician. This image was paired with a written treatment and diagnosis of gastroenteritis alongside a contradictory diagnosis and treatment plan for appendicitis from an Online Symptom Checker.
Experimental: Simulated Black Female Physician<br><br>Participants are randomized to view the clinical vignette with a simulated Black Female physician.	Behavioral: Simulated Black Female Physician<br><br>Participants in this arm of the experiment viewed one of 10 randomly selected possible images of a simulated Black Female physician. This image was paired with a written treatment and diagnosis of gastroenteritis alongside a contradictory diagnosis and treatment plan for appendicitis from an Online Symptom Checker.
Experimental: Simulated White Male Physician<br><br>Participants are randomized to view the clinical vignette with a simulated White Male physician.	Behavioral: Simulated White Male Physician<br><br>Participants in this arm of the experiment viewed one of 10 randomly selected possible images of a simulated White Male physician. This image was paired with a written treatment and diagnosis of gastroenteritis alongside a contradictory diagnosis and treatment plan for appendicitis from an Online Symptom Checker.
Experimental: Simulated White Female Physician<br><br>Participants are randomized to view the clinical vignette with a simulated White Female physician.	Behavioral: Simulated White Female Physician<br><br>Participants in this arm of the experiment viewed one of 10 randomly selected possible images of a simulated White Female physician. This image was paired with a written treatment and diagnosis of gastroenteritis alongside a contradictory diagnosis and treatment plan for appendicitis from an Online Symptom Checker.

Outcome Measures

Primary Outcome Measure:

1. Patient Confidence [Time Frame: Measured directly after viewing clinical vignette]
    a. "How confident are you that this doctor made the correct diagnosis?" [not at all confident (1) to completely confident (5)]*
    b. "How confident are you that this doctor made the correct treatment plan?" [not at all confident (1) to completely confident (5)]*
        - The Patient Confidence outcome for each study participant was the unweighted average of their ratings on questions a and b. In Study 1, this item was measured using 0-100 point scales. In Study 2, this outcome was measured using 5 point scales (as above). For all analyses, these Patient Confidence outcomes from a and b were rescaled to match the 1-5 point range from Study 2.

2. Believed Symptom Checker over Doctor [Time Frame: Measured directly after viewing clinical vignette]

    "Which diagnosis do you think is more likely to be correct?" [the doctor's diagnosis (0); online symptom checker (1)]

3. Likelihood of Requesting more Tests [Time Frame: Measured directly after viewing clinical vignette]

    "Would you ask the doctor to perform additional diagnostic tests? (Such as the CT scan recommended by the Symptom Checker)." [definitely not (1); probably not (2); might or might not (3); probably (4); definitely (5)]

4. Patient Satisfaction [Time Frame: Measured directly after viewing clinical vignette]

   "What number would you use to rate your care during this emergency room visit?" [0 (worse possible care) to 10 (best possible care)]*

   *In Study 1, this Patient Satisfaction was item measured using a 0-100 point scale. In Study 2, this was measured using a 10 point scale (as above). For all analyses, this Patient Satisfaction outcome from Study 1 was rescaled to match the 0-10 point range in Study 2.

5. Likelihood to Recommend [Time Frame: Measured directly after viewing clinical vignette]

   "Would you recommend this doctor to your friends and family?" [definitely not (1); probably not (2); might or might not (3); probably (4); definitely (5)]

6. Composite outcome [Time Frame: Created after data was collected]

   We chose to report a composite measure of the five main outcomes for ease of interpretation and reporting. Psychometric analyses (Cronbach's alpha) conducted on our outcomes indicated a high degree of internal reliability. Combining multiple items to create a single scale reduces measurement error, which increases statistical precision when estimating treatment effects.

   We created the composite outcome by extracting the first principal component from a principal component analysis and creating an index. This is a standard technique for dimension reduction when combining multiple correlated measures of the same latent factor (e.g. patient confidence and satisfaction).

Secondary Outcome Measures:

1. Warmth and Competence   [Time Frame: Measured directly after viewing clinical vignette]
    a. Study 1: "How do you imagine this doctor would be in a real interaction?". 7-item scale: Tolerant, Warm, Sincere, Good-natured, Intelligent, Competent, Confident.
    b. Study 2: "Based on the doctor's diagnosis, to what extent do you find [him/her]". 6-item scale: Kind, Qualified, Intelligent, Competent, Open-minded, Trustworthy.
        - Warmth and Competence were measured in Study 1 (7-item scale) and Study 2 (6-item scale).

2. Willingness to sue or complain   [Time Frame: Measured directly after viewing clinical vignette]

    "You take the doctor's advice and go home. Over the next few days, the pain in your abdomen got worse and you returned to the hospital where you were diagnosed with appendicitis. Your appendix had burst and you developed a serious infection. This required emergency surgery and an extended stay in the hospital's intensive Care Unit"*

    a. "Would you file a complaint against this doctor?" [5 = "Definitely"; 4 = "Probably"; 3 = "Might or might not"; 2 = "Probably not"; 1 = "Definitely not"]
    b. "Would you consider suing this doctors?" [5 = "Definitely"; 4 = "Probably"; 3 = "Might or might not"; 2 = "Probably not"; 1 = "Definitely not"]
        - Willingness to sue or complain was only measured in Study 2.

3. Fairness of the cost   [Time Frame: Measured directly after viewing clinical vignette]

"You would be charged about $350 for this emergency department visit. How fair do you think this charge is?" [0 = "Completely unfair" to 100 = "Completely Fair"]*

*Fairness of the cost was only measured in Study 1.

Eligibility Criteria

Ages Eligible for Study:	18 Years and older
Sexes Eligible for Study:	All
Gender Based:	No
Accepts Healthy Volunteers:	Yes

Criteria

Inclusion Criteria:

- Adults over 18 years old

Exclusion Criteria:

- Participants who reported current pregnancy
- Participants who reported a current or prior diagnosis of cancer
- Participants who reported a history of abdominal surgery

Contacts and Locations

Locations

United States, Connecticut

Yale University

New Haven, Connecticut, United States, 06520

Study Documents (Full-Text)

Documents provided by Yale University

Statistical Analysis Plan [PDF] March 13, 2018

More Information

Responsible Party:	Yale University
ClinicalTrials.gov Identifier:	
Other Study ID Numbers:	HIC 2000022317<br>12916<br>9068
Last Verified:	November 2019

Individual Participant Data (IPD) Sharing Statement:

Plan to Share IPD:	Yes
Plan Description:	We will make the data available upon individual request.
	More information provided by Yale University
Supporting Materials:	Study Protocol<br>Statistical Analysis Plan (SAP)<br>Informed Consent Form (ICF)<br>Analytic Code
Time Frame:	All data and replication code will be posted on an open-source website after the manuscript is accepted.
Access Criteria:	We have no restrictions on access. All data and replication code will be posted on an open-source website after the manuscript is accepted.

Human Subjects Protection Review Board Status: Exempt

Studies a U.S. FDA-regulated Drug Product: No

Studies a U.S. FDA-regulated Device Product: No
