# Supplementary material for: Effect of Physician Gender and Race on Simulated Patients’ Ratings and Confidence in Their Physicians: A Randomized Trial
Source: JAMA Netw Open. 2020 Feb 21;3(2):e1920511. doi: 10.1001/jamanetworkopen.2019.20511 (PMC7043197; doi:10.1001/jamanetworkopen.2019.20511)
Supplement: Supplement 2. — eMethods 1. Randomization and Estimation Procedures eMethods 2. Additional Design Details eMethods 3. Covariate Balance eMethods 4. Primary Outcomes eMethods 5. Survey Measures of Racial Prejudice and Sexism eMethods 6. BART Estimated Treatment Effects eMethods 7. Secondary Outcomes eFigure 1. Scenario Instructions 1 of 2 eFigure 2. Attention Check Drag and Drop (With Correct Responses Displayed) eFigure 3. Scenario Instructions 2 of 2 eFigure 4. Patient Confidence Measure in Study 1 eFigure 5. Patient Satisfaction Measure in Study 1 eFigure 6. Patient Confidence Measure in Study 2 eFigure 7. Patient Satisfaction Measure in Study 2 eFigure 8. Warmth and Competence in Study 1 eFigure 9. Warmth and Competence in Study 2 eFigure 10. Fairness of Visit in Study 1 eFigure 11. Willingness to Publish Doctor Error in Study 2 eFigure 12. Example of Explicit Prejudice Survey Item Used in Qualtrics eFigure 13. Distribution of Scores on Prejudice Items in Study 1 eFigure 14. Distribution of Scores on Prejudice Items in Study 2 eFigure 15. Distribution of Scores on Sexism Items in Study 1 eFigure 16. Distribution of Scores on Hostile Sexism Items in Study 2 eFigure 17. Distribution of Scores on Benevolent Sexism Items in Study 2 eTable 1. Background Characteristics by Treatment Group in Study 1 eTable 2. Background Characteristics by Treatment Group in Study 2 eTable 3. Randomization Inference (RI) for Covariate Balance eTable 4. Average Patient Evaluation Scores on Composite Index of Primary Outcomes eTable 5. Estimated Treatment Effects on Primary Outcomes in Combined Sample eTable 6. Estimated Treatment Effects on Primary Outcomes in Study 1 eTable 7. Estimated Treatment Effects on Primary Outcomes in Study 2 eTable 8. Summary Statistics for BART-Estimated Treatment Effects on Composite Index (0-100) eTable 9. Estimated Treatment Effects on Secondary Outcomes in Study 1 eTable 10. Estimated Treatment Effects on Secondary Outcomes in Study 2 eReferences. [file jamanetwopen-3-e1920511-s002.pdf]

Supplementary Online Content

Solnick RE, Peyton K, Kraft-Todd G, Safdar B. Effect of physician gender and race on simulated patients' ratings and confidence in their physicians: a randomized trial. *JAMA Netw Open.* 2020;3(2):e1920511. doi:10.1001/ jamanetworkopen.2019.20511

eMethods 1. Randomization and Estimation Procedures.

eMethods 2. Additional Design Details

eMethods 3. Covariate Balance

eMethods 4. Primary Outcomes

eMethods 5. Survey Measures of Racial Prejudice and Sexism

eMethods 6. BART Estimated Treatment Effects

eMethods 7. Secondary Outcomes

eFigure 1. Scenario Instructions 1 of 2

eFigure 2. Attention Check Drag and Drop (with Correct Responses Displayed)

eFigure 3. Scenario Instructions 2 of 2

eFigure 4. Patient Confidence Measure in Study 1

eFigure 5. Patient Satisfaction Measure in Study 1

eFigure 6. Patient Confidence Measure in Study 2

eFigure 7. Patient Satisfaction Measure in Study 2

eFigure 8. Warmth and Competence in Study 1

eFigure 9. Warmth and Competence in Study 2

eFigure 10. Fairness of Visit in Study 1

eFigure 11. Willingness to Publish Doctor Error in Study 2

eFigure 12. Example of Explicit Prejudice Survey Item Used in Qualtrics

eFigure 13. Distribution of Scores on Prejudice Items in Study 1

eFigure 14. Distribution of Scores on Prejudice Items in Study 2

eFigure 15. Distribution of Scores on Sexism Items in Study 1

eFigure 16. Distribution of Scores on Hostile Sexism Items in Study 2

eFigure 17. Distribution of Scores on Benevolent Sexism Items in Study 2

eTable 1. Background Characteristics by Treatment Group in Study 1

eTable 2. Background Characteristics by Treatment Group in Study 2

eTable 3. Randomization Inference (RI) for Covariate Balance

eTable 4. Average Patient Evaluation Scores on Composite Index of Primary Outcomes

eTable 5. Estimated Treatment Effects on Primary Outcomes in Combined Sample

eTable 6. Estimated Treatment Effects on Primary Outcomes in Study 1

© 2020 Solnick RE et al. *JAMA Network Open.*

eTable 7. Estimated Treatment Effects on Primary Outcomes in Study 2

eTable 8. Summary Statistics for BART Estimated Treatment Effects on Composite Index (0-100)

eTable 9. Estimated Treatment Effects on Secondary Outcomes in Study 1

eTable 10. Estimated Treatment Effects on Secondary Outcomes in Study 2

eReferences.

This supplementary material has been provided by the authors to give readers additional information about their work.

1 Randomization and Estimation Procedures

The randomization scheme in this experiment is a two step process for each subject. First, one of four race/gender pairs is drawn from a uniform distribution:

$$f(Z = z) = \begin{cases} 0.25 & : z \in \{BF, BM, WF, WM\} \\ 0 & : o.w. \end{cases}$$

Next, conditional on the realized value of Z, one of 10 potential doctors is sampled from that group:

$$f(K = k|Z = z) = \begin{cases} 0.10 & : k \in \{1, 2, \ldots, 10\}, z = BF \\ 0.10 & : k \in \{1, 2, \ldots, 10\}, z = BM \\ 0.10 & : k \in \{1, 2, \ldots, 10\}, z = WF \\ 0.10 & : k \in \{1, 2, \ldots, 10\}, z = WF \\ 0 & : o.w. \end{cases}$$

For example, $f(K = 10|Z = BF) = 0.10$ is the probability that doctor #10 is selected, given that the doctor is a Black Female. The joint distribution of K and Z is simply,

$$f(Z = z, K = k) = \begin{cases} 0.025 & : z = BF, k \in \{1, 2, \ldots, 10\} \\ 0.025 & : z = BM, k \in \{1, 2, \ldots, 10\} \\ 0.025 & : z = WF, k \in \{1, 2, \ldots, 10\} \\ 0.025 & : z = WM, k \in \{1, 2, \ldots, 10\} \\ 0 & : o.w. \end{cases}$$

We treat all potential realizations of doctor race and gender as exchangeable, so we do not distinguish between, e.g. Black Female #1 and Black Female #2. For example, let $f(BF_{\{1\}}, \ldots, BF_{\{10\}})$ denote the joint density of each of 10 possible Black Female doctors, e.g. BF_9 is "Black Female doctor number 9". Exchangeability assumes that $f(BF_{\{1\}}, \ldots, BF_{\{10\}}) = f(BF_{\{\pi_1\}}, \ldots, BF_{\{\pi_{10}\}},)$ for all permutations π of $\{1, \ldots, 10\}$. In other words, the subscript conveys no information so that, for example, Black Female #7 and Black Female #1 are "exchangeable". Exchangeability follows from the random assignment process since, conditional on $W = BF$, $BF_{\{1\}}, \ldots, BF_{\{10\}}$ are independent and identically distributed.

Since we are invoking this simplifying assumption, each subject i has just 4 potential outcomes (one for each level of $Z \in \{BF, BM, WF, WM\}$) for a given outcome variable Y, i.e. $Y_i(Z = BF), Y_i(Z = BM), Y_i(Z = WF), Y_i(Z = WM)$. Let $\tau_i^{\text{BF}} := Y_i(BF) - Y_i(WM)$ denote the individual level treatment effect of the Black Female doctor, relative to the White Male, $\tau_i^{\text{BM}} := Y_i(BM) - Y_i(WM)$ denote the effect of the Black Male doctor, and $\tau_i^{\text{WF}} := Y_i(WF) - Y_i(WM)$ denote the effect of the White Female doctor. Thus, for each individual we have three potential "treatment contrasts", since White Male is the "control". More generally, an experiment with J conditions has $\frac{J(J-1)}{2}$ possible comparisons (where $J = 4$ in this design, for 6 possible pairwise comparisons).

© 2020 Solnick RE et al. *JAMA Network Open.*

Since we are fixing the "control" to be White Male (per our pre-analysis plans), then we just have three target parameters for a given outcome variable (rather than six): $\tau^{BF}, \tau^{BM}, \tau^{WF}$. Each target parameter is an "average treatment contrast". For a given individual, we only observe one of the 4 potential outcomes. An unbiased estimator for each average treatment contrast, and a given outcome variable Y, is simply the difference in means:

$$\hat{\tau}^{\mathrm{BF}} = \frac{1}{N_{BF}} \sum_{i=1}^{N} \mathbb{1}\left\{Z_i = BF\right\} Y_i - \frac{1}{N_{WM}} \sum_{i=1}^{N} \mathbb{1}\left\{Z_i = WM\right\} Y_i$$
$$\hat{\tau}^{\mathrm{BM}} = \frac{1}{N_{BM}} \sum_{i=1}^{N} \mathbb{1}\left\{Z_i = BM\right\} Y_i - \frac{1}{N_{WM}} \sum_{i=1}^{N} \mathbb{1}\left\{Z_i = WM\right\} Y_i$$
$$\hat{\tau}^{\mathrm{WF}} = \frac{1}{N_{WF}} \sum_{i=1}^{N} \mathbb{1}\left\{Z_i = WF\right\} Y_i - \frac{1}{N_{WM}} \sum_{i=1}^{N} \mathbb{1}\left\{Z_i = WM\right\} Y_i$$

where N_{BF} denotes the number of subjects that receive the Black Female treatment, $N = N_{BF} + N_{BM} + N_{WF} + N_{WM}$, $\mathbb{1}\left\{Z_i = BF\right\}$ is an indicator function that takes on a value 1 if subject i received the Black Female treatment and zero otherwise, and Y_i is a vector of outcomes for all subjects in the sample ($i = 1, \ldots, N$).

Linear regression of Y_i on Z_i is a straightforward estimator, where Z_i is a 4 level factor with WM as the "omitted category" and the $4 - 1$ coefficients (excluding the intercept) provide unbiased estimates of each treatment contrast. This is equivalent to a linear regression of Y_i on three indicator variables: $Z_i^{BF} = \mathbb{1}\{Z_i = BF\}$, $Z_i^{BM} = \mathbb{1}\{Z_i = BM\}$, and $Z_i^{WF} = \mathbb{1}\{Z_i = WF\}$. This is also equivalent to three "difference-in-means" comparisons where the "control group" observations are those that received the White Male treatment.

The covariate-adjusted difference in means estimator is simply the difference-in-means estimator adjusted for differences in background characteristics to improve precision (*1*). All estimates of treatment contrasts presented here, and in the manuscript, are from the covariate-adjusted difference-in-means estimator.

© 2020 Solnick RE et al. *JAMA Network Open.*

2 Additional Design Details

Summary of Study Procedures:

1. **Background Questions**: Participants responded to background questions related to healthcare (self-assessed health, trust in doctors, insurance, etc.)
2. **Vignette Instructions**: Participants read the scenario describing their role as a patient presenting to an Emergency Department (ED) with abdominal pain.
3. **Attention Check**: Participants completed attention check questions to ensure understanding of the vignette and recall of case details.
4. **Treatment Assignment**: Random assignment to one of four treatment arms of physician identity (Black Female, Black Male, White Female, or White Male). Random assignment to one of 10 images, created from Chicago Face Database (CFD).
5. **Clinical Vignette**: Shown image of the physician and the physician's diagnosis and treatment plan alongside a contradictory diagnosis and treatment plan from an Online Symptom Checker.
6. **Outcome Measurement**: Primary and secondary outcomes measured.
7. **Additional Covariates**: Subject responded to questions about racial prejudice and sexism

Prior to participating in the clinical vignette, subjects were provided with a description of their symptoms (Figure e1). The description was displayed for 30 seconds while the "continue" button was disabled so that each subject was required to spend at least 30 seconds reviewing the instructions. Next, the subject was asked two questions as part of an "attention check" procedure (*2*, *3*). The first question simply asked "How long have you been experiencing abdominal pain?" with potential responses "For a couple of hours"; "For about one day" (correct answer); or "Weeks". Next, the subject completed the "drag and drop" task in Figure e2. If the subject passed both attention check questions they were advanced to a new page and provided with additional instructions about the task (Figure e3). If the subject failed, they were re-directed to the scenario instructions for a second review. In Study 1, 82% of subjects passed on the first attempt; in Study 2, 57% passed on the first attempt. The significant difference in pass rates between the MTurk (Study 1) and Lucid samples (Study 2) is consistent with prior research finding MTurk workers are substantially more attentive to instructions than student research subjects, or research subjects drawn from the general population (*4*, *5*). None of the subjects who failed the attention check in these studies were excluded from analyses reported here, or in the manuscript (*6*).

Selection of Simulated Physicians

Selection criteria of images within each treatment arm was based on CFD, raters' evaluations along the following dimensions: 1) perceived age between, 27 and 39 years old, the group of physicians more likely to experience discrimination (*7*); 2) 90% agreement among raters on perceived race and gender of face; 3) perceived trustworthiness and attractiveness between 3-5 on a 7-point Likert scale, excluding those perceived to be unusually trustworthy (or untrustworthy) or unusually attractive (or unattractive).

© 2020 Solnick RE et al. *JAMA Network Open.*

3 Covariate Balance

Tables e1 and e2 show summary statistics for background covariates across all four treatment arms. One implication of random assignment is that background characteristics should be poor predictors of treatment assingment. Rather than conducting separate tests covariate-by-covariate, for each treatment arm, and each study, we use randomization inference to conduct two omnibus tests of the null hypothesis of covariate balance across treatment arms (see Chapter 3 of (*8*) for a textbook treatment). This test is performed by regressing the treatment assignment vector on background covariates. When the null hypothesis of covariate balance is true, the observed F-statistic from this regression will not be unusual when compared to the null distribution implied by the experimental design.

We approximate the null distribution using 10,0000 permutations of the experimental design. The RI P-value is then the proportion of permuted F-statistics that are as extreme as the one observed under the null hypothesis of covariate balance, i.e. if none of the permuted F-statistics were as extreme as the one observed then the P-value would be zero, providing strong evidence of covariate imbalance. The observed estimates, along with the 0.025th and 0.975th quantiles of the distribution of permuted estimates and the RI P-values, are presented for each Study in Table e3.

The RI P-value for Study 1 is 0.90; that is, approximately 90% of the simulated F-statistics as extreme as the observed F-statistic of 0.53. The RI p-value for Study 1 is 0.18; that is, approximately 18% of the simulated F-statistics were as extreme as the observed F-statistic of 1.35. Thus, we fail to reject the null hypothesis of covariate balance in both Study 1 and Study 2, as implied by the experimental designs.

© 2020 Solnick RE et al. *JAMA Network Open.*

4 Primary Outcomes

The primary outcome measures used in Study 1 and Study 2 are enumerated below:

1) *Patient Confidence*: 1a) "How confident are you that this doctor made the correct diagnosis?" 1b) "How confident are you that this doctor recommended the correct treatment plan?"
2) *Believes Symptom Checker*: "Which diagnosis do you think is more likely to be correct?" [1 = "The symptom checker"; 0 = "The doctor"].
3) *Requests more tests*: "Would you ask the doctor to perform additional diagnostic tests? (Such as the CT scan recommended by the Symptom Checker)." [5 = "Definitely"; 4 = "Probably"; 3 = "Might or might not"; 2 = "Probably not"; 1 = "Definitely not"]
4) *Patient Satisfaction*: "What number would you use to rate your care during this emergency room visit?"
5) *Likelihood to Recommend*: "Would you recommend this doctor to your friends and family?" [5 = "Definitely"; 4 = "Probably"; 3 = "Might or might not"; 2 = "Probably not"; 1 = "Definitely not"]

The patient confidence outcome for each study participant is simply the unweighted average of their ratings on question 1a and 1b. All other primary outcomes are based on a single survey item. In Study 1, outcomes 1a, 1b and 4 were measured using 0-100 point scales (Figure e4-e5). In Study 2, outcomes 1a and 1b were measured using 5 point scales, and outcome 4 was measured using a 10 point scale (Figure e6-e7). For all analyses, outcomes 1a and 1b from Study 1 are simply rescaled to match the 1-5 point range for 1a and 1b in Study 2. Likewise, outcome 4 from Study 1 is rescaled to match the 0-10 point range in Study 2. For all analyses, outcomes 2-3 are rescaled so that higher values correspond to more positive evaluations of physician: *Believes Symptom Checker* (0 ["the doctor"], 1 ["the symptom checker"]); *Requests more tests* (1 ["Definitely"] to 5 ["Definitely not"]). Table e5 presents the estimated treatment effects for all primary outcome variables when pooled across both studies. Table e6 presents the estimated treatment effects for all primary outcomes for Study 1 only. Table e7 presents the analoguous results for Study 2 only.

5 Survey Measures of Racial Prejudice and Sexism

In Study 1 and Study 2, the measure of racial prejudice is an explicit (survey based) measure that captures negative beliefs about group-level differences between blacks and whites on four dimensions: trustworthiness, violence, work-ethic and intelligence (*9*, *10*). We scale responses for each of the 4 individual items (e.g. Figure e12 shows the "trustworthiness item") so that a positive difference for "whites" versus "blacks" indicates belief in group-level white superiority. The White-Black differences for each of the items are summarized in Figure e13 (for Study 1), and Figure e14 (for Study 2). The modal respondent – in both studies – did not endorse group-level differences between "blacks" and "whites". We combined these individual items into a single measure by summing across all 4 items to create a racial prejudice index with range -24 to 24. Approximately 40% of subjects in Study 1 and 34% of subjects in Study 2 scored above zero on the index, and therefore believed in the group-level superiority of whites over blacks.

In Study 1, hostile sexism was measured using components from the ambivalent sexism inventory (*11*), each with a 6-point scale from Strongly disagree (0) to Strongly agree (5), where higher levels

 © 2020 Solnick RE et al. *JAMA Network Open.*

of agreement reflect higher levels of sexism:

1) sexism1: Women exaggerate problems they have at work.
2) sexism2: Once a woman gets a man to commit to her, she usually tries to put him on a tight leash.
3) sexism3: Women are too easily offended.
4) sexism4: Many women are actually seeking special favors, such as hiring policies that favor them over men, under the guise of asking for "equality."

The distribution of responses for each individual item is plotted in Figure e15. The modal response category, for each item, was Strongly disagree (0). We created a sexism index ranging from 1 to 6 by taking the average across the 5 individual items. In Study 2, we used a 2-dimensional measure that distinguished between hostile (2-items) and benevolent (3-items) sexism (*12*). Each individual item was captured using a 5-point scale from Strongly agree (5) to Strongly disagree (1) with a neutral midpoint of "Neither Agree nor Disagree" (3).

Hostile Sexism:

1) asi_hostile1: Women seek power by gaining control over men.
2) asi_hostile2: Once a woman gets a man to commit to her, she usually tries to put him on a tight leash.

Benevolent Sexism:

1) asi_benevolent1: Women should be cherished and protected by men.
2) asi_benevolent2: Women have a quality of purity that few men possess.
3) asi_benevolent3: Despite accomplishment, men are incomplete without women.

We constructed a hostile sexism index (range 1 to 5) and a benevolent sexism index (range 1 to 5) for each respondent by averaging across the individual items. The distribution of responses for each individual item is plotted in Figure e16 for Hostile Sexism and Figure e17 for Benevolent Sexism.

6 BART Estimated Treatment Effects

Recall that we are interested in 3 treatment contrasts: $\tau_i^{\text{BF}} := Y_i(BF) - Y_i(WM)$, $\tau_i^{\text{BM}} := Y_i(BM) - Y_i(WM)$, and $\tau_i^{\text{WF}} := Y_i(WF) - Y_i(WM)$. Rather than estimate an average treatment contrast, e.g. $\hat{\tau}^{BF}$, the Bayesian Additive Regression Trees (BART) algorithm seeks to estimate the *individual-level potential outcomes* for each unit (e.g. $Y_i(WM), Y_i(WF), Y_i(BM), Y_i(BF)$) by taking into account background covariates, and allowing for higher-order interactions between covariates and treatment. BART considers each of these individual-level responses to be a random variable, conditional on the observed data. Estimation and inference for BART proceeds by taking many draws from the posterior distribution of each potential outcome, for each individual.

We implemented the BART algorithm using the dbarts package in R, sampling 5,000 draws from the posterior distribution using Markov Chain Monte Carlo (MCMC), with 1,000 iterations of burn-in, 200 trees, 4 independent chains, and thinning every 5 iterations. Thus, for each individual we obtained $20,000 = 5,000 \times 4$ draws from the posterior distribution of each potential outcome: $Y_i(BF), Y_i(BM), Y_i(WF), Y_i(WM)$. These draws are then used to construct 20,000 estimates of $\hat{\tau}_i^{BF}$, $\hat{\tau}_i^{BM}$, $\hat{\tau}_i^{WF}$ for each individual. Point estimates and 95% credible intervals are formed by taking

© 2020 Solnick RE et al. *JAMA Network Open.*

the average and corresponding sample quantiles of the posterior distribution for each individual. The point estimates and 95% credible intervals presented in the manuscript are based on this procedure.

The Composite Index used for estimation was created by extracting the first principal component from a principal component analysis (PCA) on all primary outcome measures. In addition to the background covariates used for regression adjustment, each BART model included measures of racial prejudice and sexism, as well as an indicator for whether the subject passed the pre-treatment attention check questions about the scenario instructions on the first attempt. Table e8 provides an overall summary of the BART estimated treatment effects for each study across each of the three treatment contrasts. The first column reports the proportion of BART estimated (individual-level) treatment effects that were predicted to be positive. In none of the cases where a subject was predicted to have a positive (or negative) treatment effect does their corresponding 95% credible interval exclude zero. The second column reports the mean of the BART estimated treatment effects, the third reports the standard deviation, and the final two columns report the 0.025th and 0.975th quantiles.

7 Secondary Outcomes

The secondary outcome measures differed across Study 1 and Study 2. In Study 1, physician warmth and competence were measured using the instrument presented in Figure e8 (*13*, *14*). In Study 2, physician warmth and competence were measured using the instrument presented in Figure e9 (*15*). Warmth and competence scales in Study 1 and Study 2 were constructed by taking the first principal component from a PCA on all individual scale items. The fairness of the ER visit was measured using the instrument in Figure e10, and the willingness to punish was measured using the instrument in Figure e11. Table e9 presents the estimated treatment effects for all secondary outcome variables measured in Study 1. Table e10 presents the analoguous results for Study 2.

© 2020 Solnick RE et al. *JAMA Network Open.*

8 Figures e1 to e18

Figure e1: Scenario Instructions 1 of 2

Thank you for completing the background questions! We will now move to the main part of the study. In this part, imagine you are a patient in an interaction with an emergency medicine doctor at a hospital.

Please carefully read the scenario below, as you will be asked to enter your symptoms on the interface at the next page.

You have been experiencing **abdominal pain since yesterday**. The pain has been slowly **getting worse over the last 24 hours**. It is a **cramping pain** that feels the worst around your belly button area. You haven't felt hungry since the pain started. You have experienced **nausea and vomiting**. Although you weren't able to keep down your last meal, you tried drinking some water and were able to keep that down.

Most recently, you vomited clear liquid. You have also had **three episodes of watery diarrhea** in the last 24 hours. There was no blood in the diarrhea. You **do not have a fever**, and haven't been camping or traveling recently. You decide to seek medical attention in the emergency department of a hospital.

© 2020 Solnick RE et al. *JAMA Network Open.*

Figure e2: Attention Check Drag and Drop (with correct responses displayed)

Which of the following symptoms have you been experiencing?

Please drag and drop the symptoms that **you have been experiencing** to the box titled "my symptoms", and drag the symptoms that you **have not been experiencing** to the "not my symptoms" box.

Items

my symptoms	not my symptoms
1 Abdominal pain	1 Cough
2 Diarrhea	2 Throat irritation
	3 Chest pain
	4 Headache

Figure e3: Scenario Instructions 2 of 2

While waiting to see the doctor you research your symptoms on the internet by entering them into an online "Symptom Checker" (for example, WebMD or Mayo Clinic). The Symptom Checker provides you with a list of diseases and conditions that match what you reported.

We have entered these symptoms into a real symptom checker and received a diagnosis. We have also asked real emergency medicine doctors to make a diagnosis based on these symptoms. You will see the diagnosis from one of these doctors on the next page, along with the diagnosis provided by the Symptom Checker. Please carefully read both diagnoses and answer the questions that follow.

Please note: the next page will contain an image of the doctor, and depending on your web browser and internet connection it may take a few moments to load. The advance (>>) button will not appear until the next page is ready to view. Thank you for your patience.

© 2020 Solnick RE et al. *JAMA Network Open.*

Figure e4: Patient Confidence Measure in Study 1

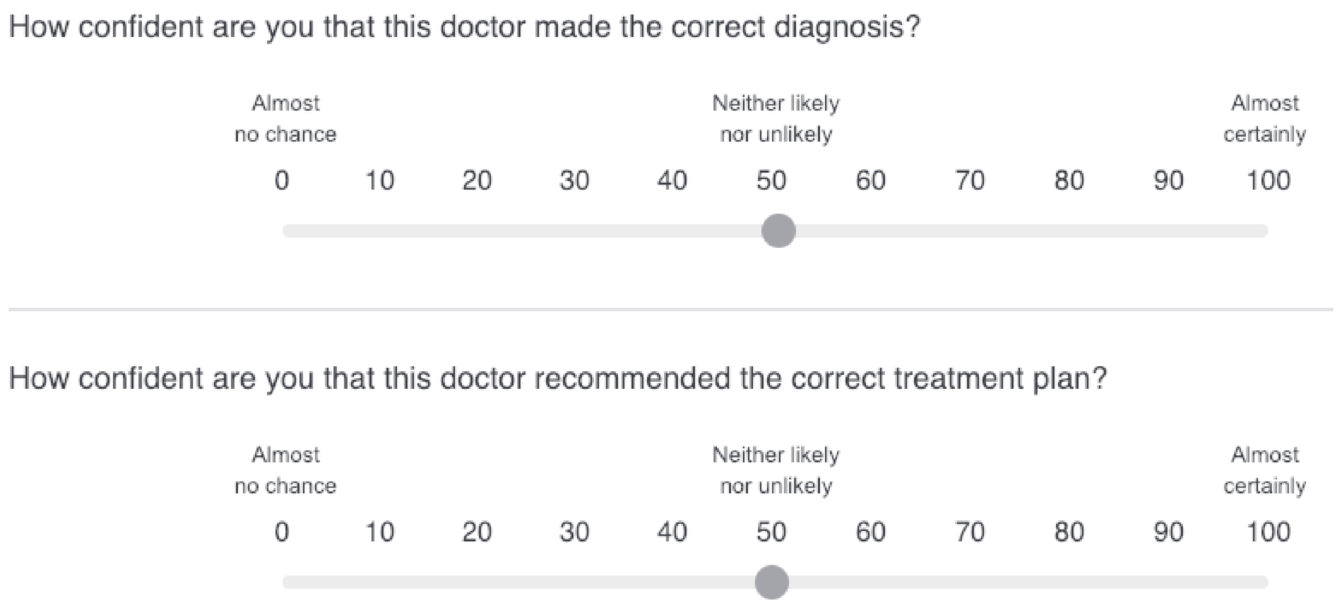

Figure e5: Patient Satisfaction Measure in Study 1

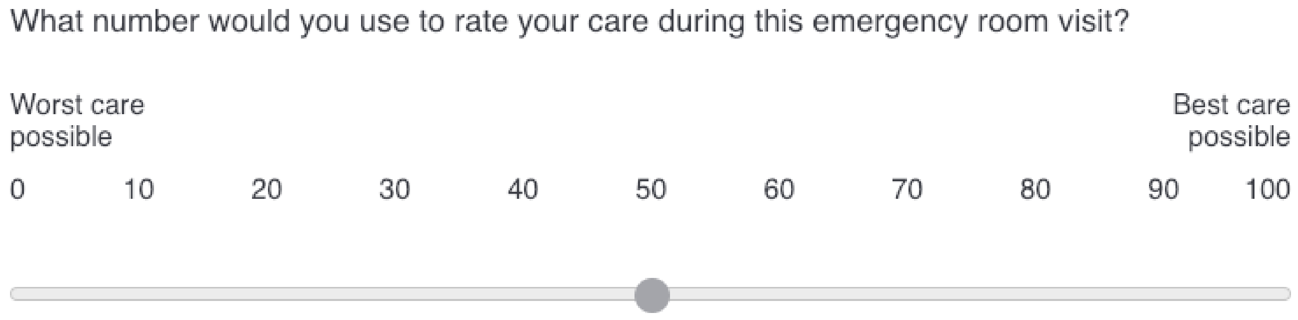

© 2020 Solnick RE et al. *JAMA Network Open.*

Figure e6: Patient Confidence Measure in Study 2

How confident are you that this doctor made the correct **diagnosis**?

Not at all confident	Slightly confident	Moderately confident	Very confident	Extremely confident
O	O	O	O	O

How confident are you that this doctor recommended the correct **treatment plan**?

Not at all confident	Slightly confident	Moderately confident	Very confident	Extremely confident
O	O	O	O	O

Figure e7: Patient Satisfaction Measure in Study 2

What number would you use to rate your care during this emergency room visit?

Worst care possible — Best care possible

0	1	2	3	4	5	6	7	8	9	10
O	O	O	O	O	O	O	O	O	O	O

© 2020 Solnick RE et al. *JAMA Network Open.*

Figure e8: Warmth and Competence in Study 1

How do you imagine this doctor would be in a real interaction?

	Not at all	Slightly	Moderately	Very	Extremely
Tolerant	○	○	○	○	○
Warm	○	○	○	○	○
Sincere	○	○	○	○	○
Good-natured	○	○	○	○	○
Intelligent	○	○	○	○	○
Competent	○	○	○	○	○
Confident	○	○	○	○	○

Figure e9: Warmth and Competence in Study 2

Based on this doctor's diagnosis, to what extent do you find him...

Unkind						Kind
○	○	○	○	○	○	○
Unqualified						Qualified
○	○	○	○	○	○	○
Unintelligent						Intelligent
○	○	○	○	○	○	○
Incompetent						Competent
○	○	○	○	○	○	○
Close-minded						Open-minded
○	○	○	○	○	○	○
Untrustworthy						Trustworthy
○	○	○	○	○	○	○

© 2020 Solnick RE et al. *JAMA Network Open.*

Figure e10: Fairness of Visit in Study 1

You would be charged about $350 for this emergency department visit. How fair do you think this charge is?

Completely unfair					Neither fair nor unfair					Completely fair
0	10	20	30	40	50	60	70	80	90	100

Figure e11: Willingness to Punish Doctor Error in Study 2

You take the doctor's advice and go home. Over the next few days the pain in your abdomen got worse and you returned to the hospital where you were diagnosed with appendicitis. Your appendix had burst and you developed a serious infection. This required an emergency surgery and an extended stay in the hospital's Intensive Care Unit.

Would you file a complaint against this doctor?

- Definitely
- Probably
- Might or might not
- Probably not
- Definitely not

Would you consider suing this doctor?

- Definitely
- Probably
- Might or might not
- Probably not
- Definitely not

© 2020 Solnick RE et al. *JAMA Network Open.*

Figure e12: Example of Explicit Prejudice survey item used in Qualtrics

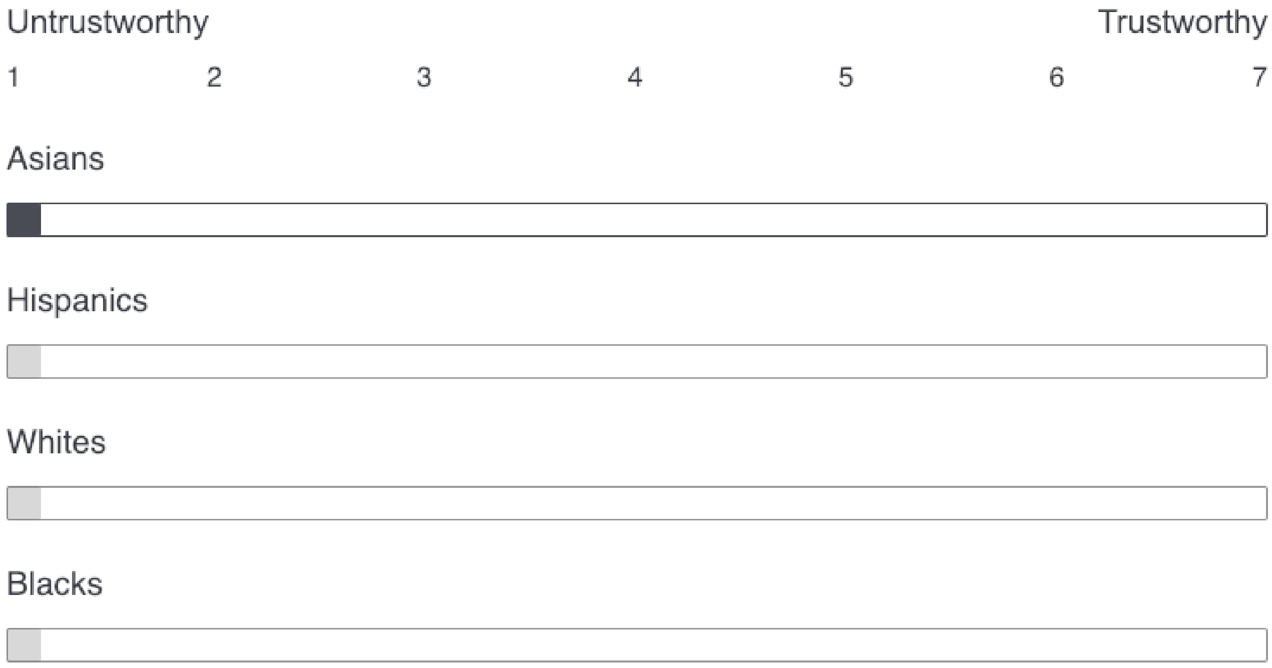

© 2020 Solnick RE et al. *JAMA Network Open.*

Figure e13: Distribution of Scores on Prejudice Items in Study 1

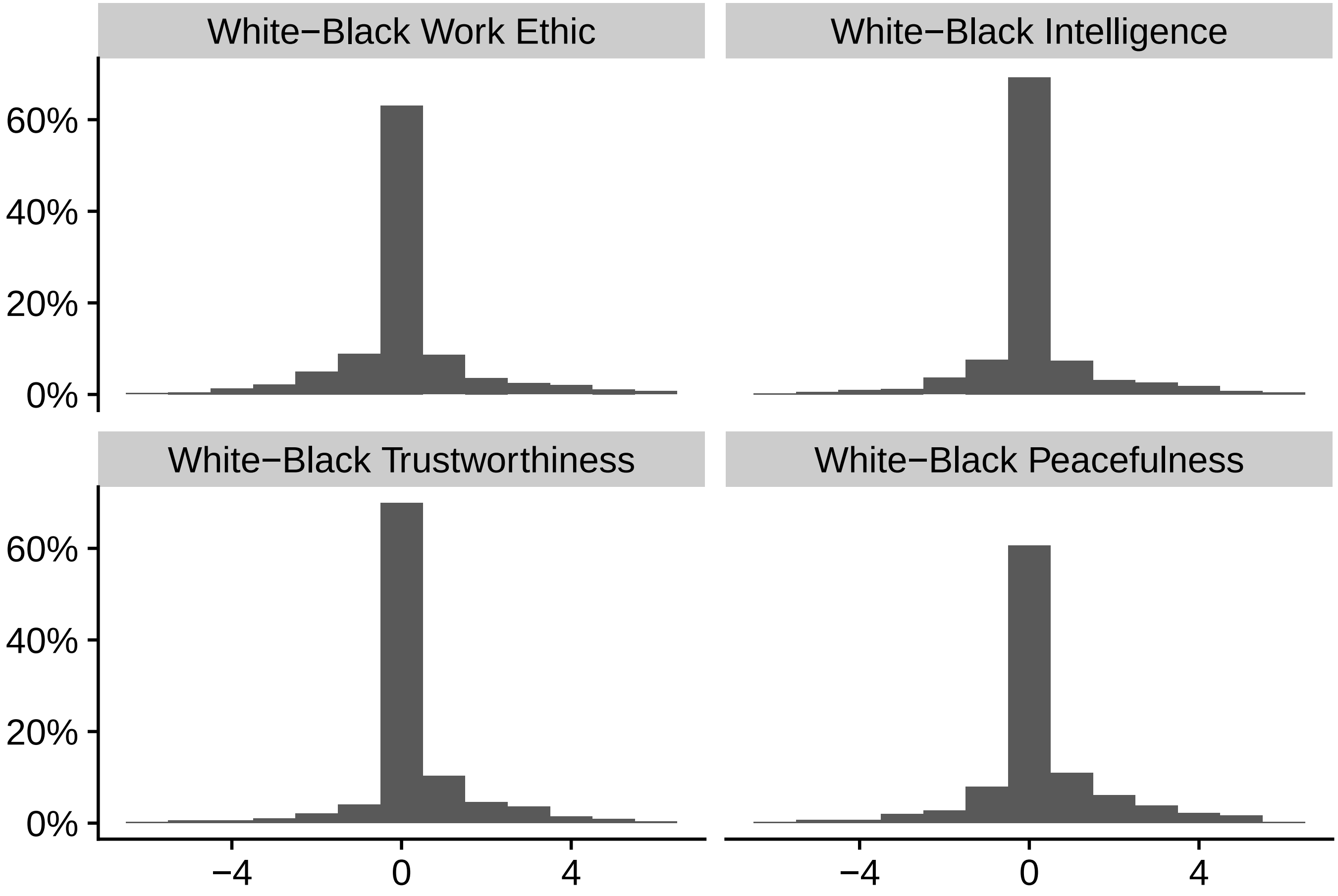

© 2020 Solnick RE et al. *JAMA Network Open.*

Figure e14: Distribution of Scores on Prejudice Items in Study 2

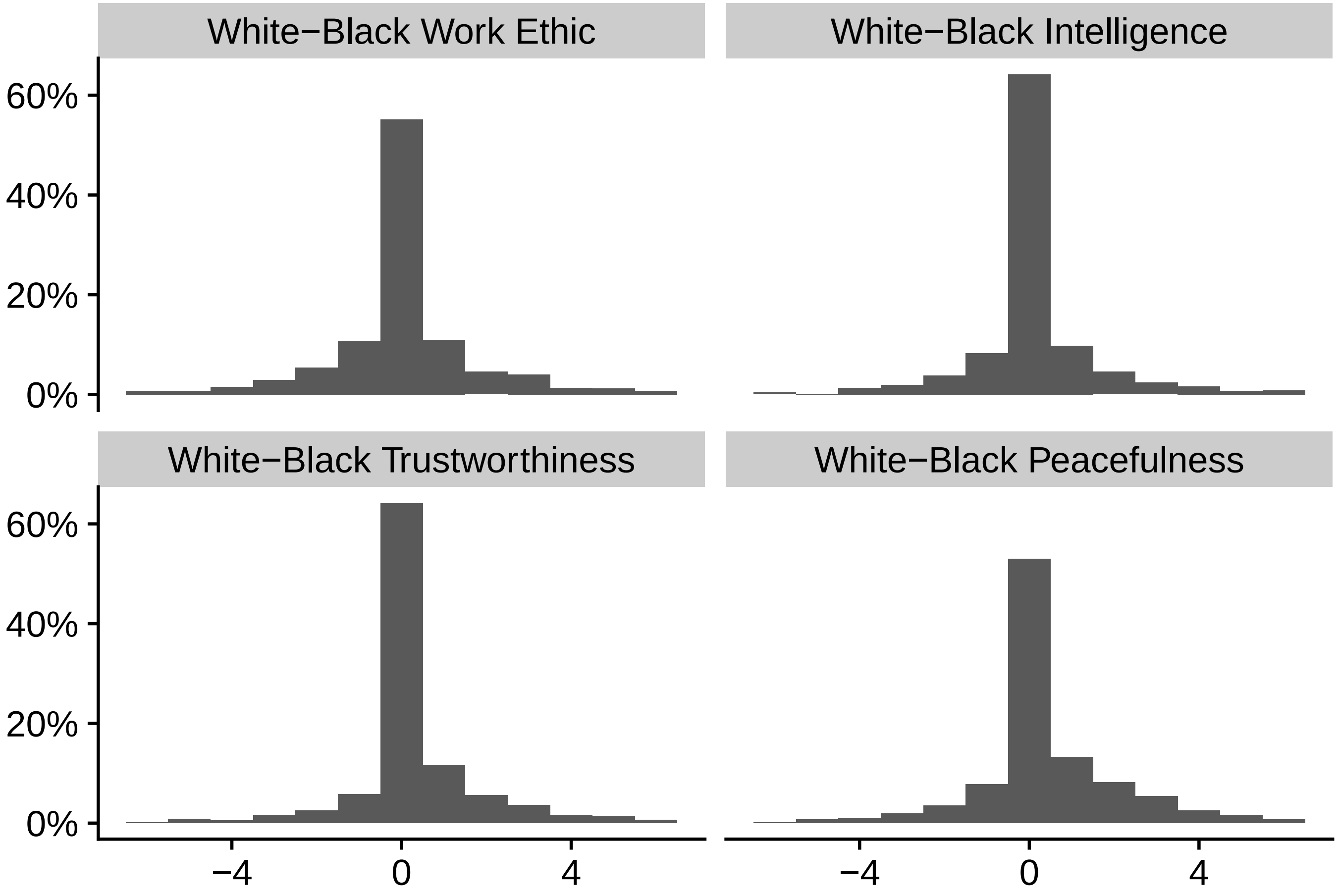

© 2020 Solnick RE et al. *JAMA Network Open.*

Figure e15: Distribution of Scores on Sexism Items in Study 1

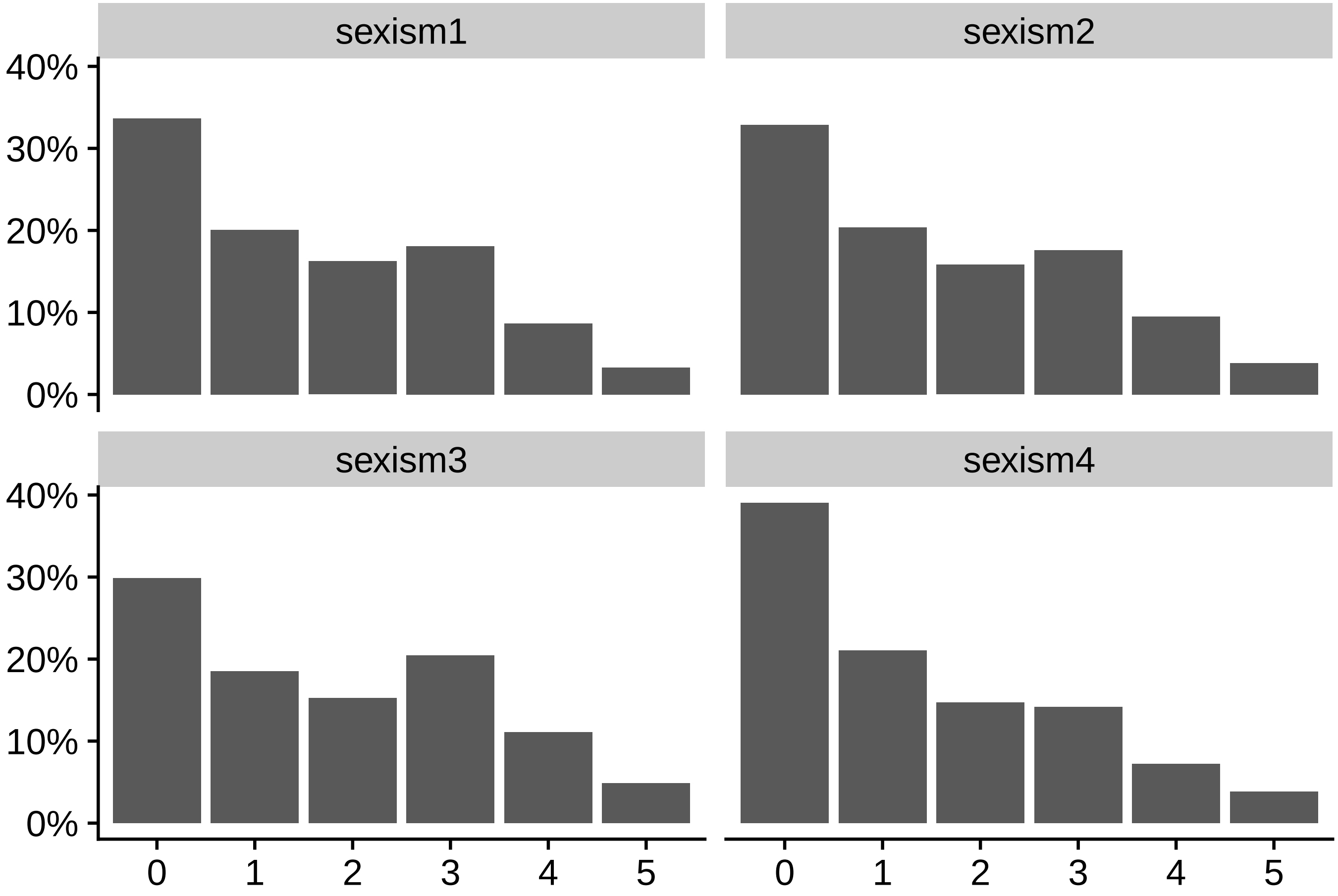

© 2020 Solnick RE et al. *JAMA Network Open.*

Figure e16: Distribution of Scores on Hostile Sexism Items in Study 2

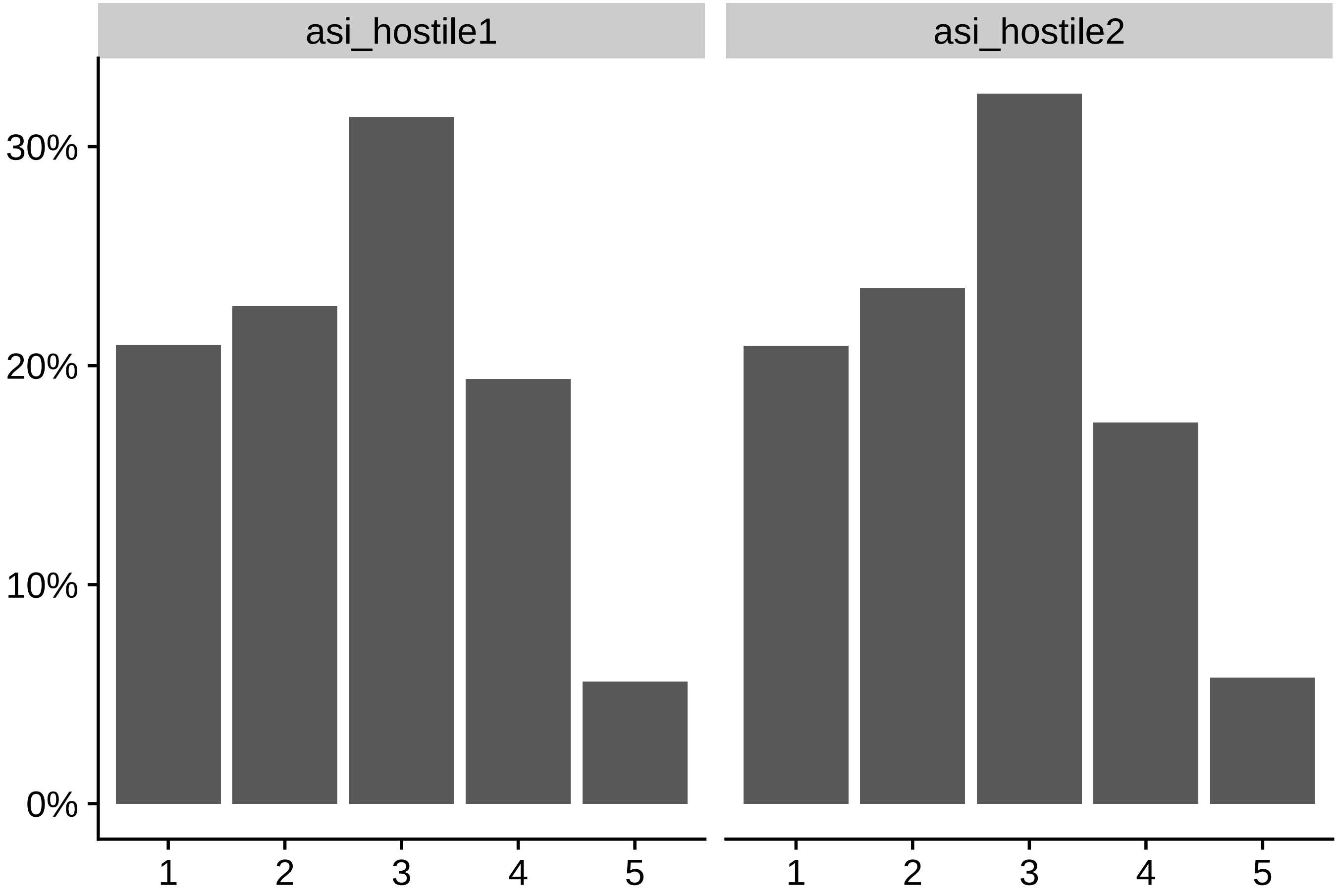

© 2020 Solnick RE et al. *JAMA Network Open.*

Figure e17: Distribution of Scores on Benevolent Sexism Items in Study 2

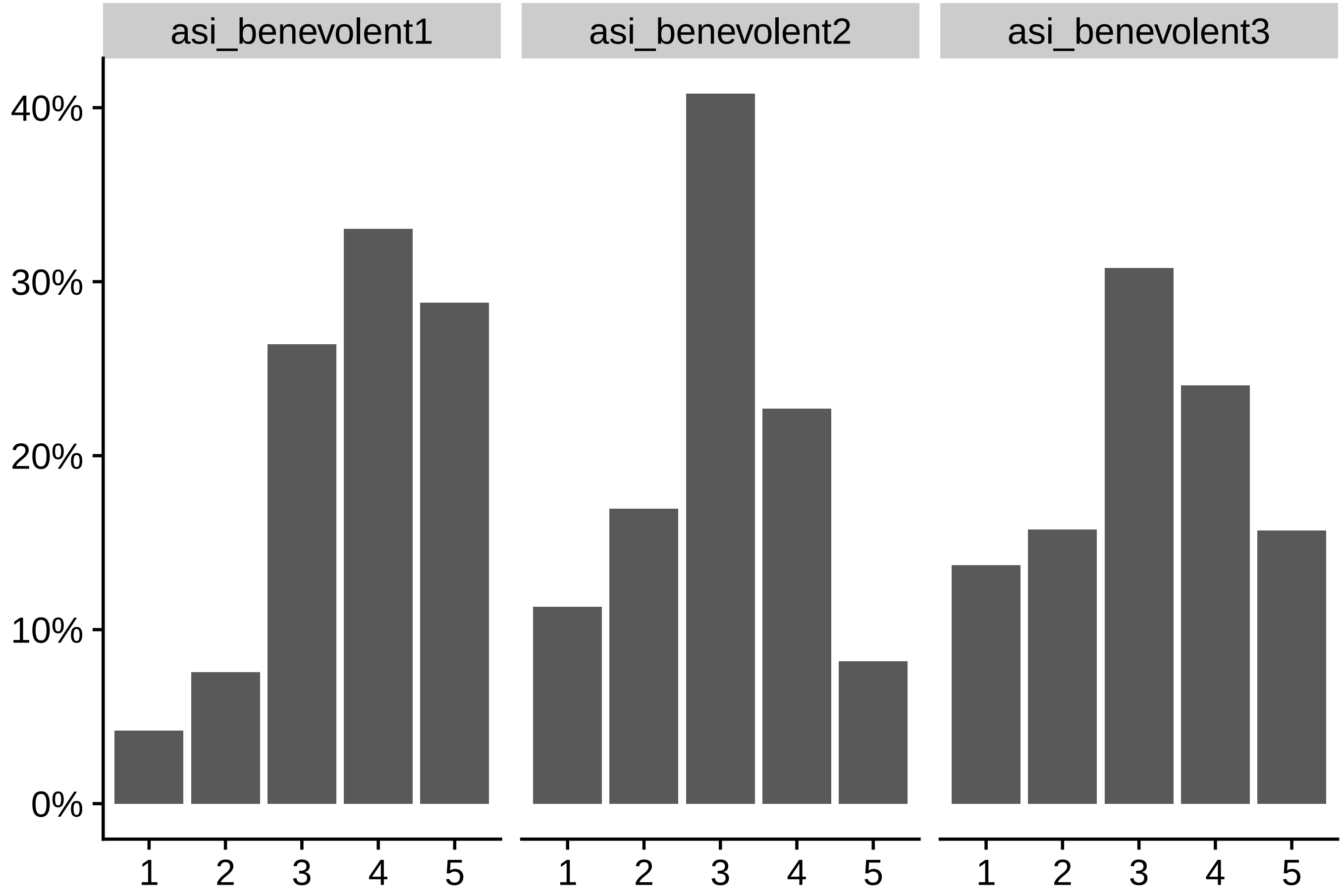

© 2020 Solnick RE et al. *JAMA Network Open.*

9 Tables e1 to e9

Table e1: Background Characteristics by Treatment Group in Study 1

	Treatment Group			
	Simulated White Male Physician	Simulated White Female Physician	Simulated Black Female Physician	Simulated Black Male Physician
Median Age (Min, Max)	50 (20,81)	50 (20,77)	50 (22,89)	50 (19,75)
Female	218 (51.78%)	241 (55.02%)	211 (54.95%)	203 (53.99%)
College Educated	217 (51.54%)	217 (49.54%)	185 (48.18%)	199 (52.93%)
HHI Below Median	245 (58.19%)	263 (60.46%)	238 (61.98%)	221 (59.25%)
**Race/Ethnicity:**				
White (Non-Hispanic)	341 (81%)	354 (80.82%)	294 (76.56%)	311 (82.71%)
Black	43 (10.21%)	43 (9.82%)	39 (10.16%)	32 (8.51%)
Hispanic	13 (3.09%)	19 (4.34%)	19 (4.95%)	13 (3.46%)
Other	24 (5.7%)	22 (5.02%)	32 (8.33%)	20 (5.32%)
**Insurance status:**				
Medicaid	7 (1.66%)	12 (2.74%)	7 (1.82%)	8 (2.13%)
Medicare	33 (7.84%)	36 (8.22%)	25 (6.51%)	27 (7.18%)
Uninsured	51 (12.11%)	60 (13.7%)	63 (16.41%)	55 (14.63%)
**Healthcare experience:**				
Unpaid Medical Bills	108 (25.65%)	109 (24.89%)	89 (23.18%)	78 (20.74%)
1+ ED visit last 6 MOs.	68 (16.15%)	69 (15.75%)	56 (14.58%)	47 (12.5%)
**Subjective assessments:**				
Mental Health Avg. (SD)	3.62 (1.12)	3.6 (1.11)	3.71 (1.09)	3.69 (1.13)
Overall Health Avg. (SD)	3.41 (0.95)	3.36 (0.92)	3.5 (0.98)	3.5 (0.94)
Trust in Docs Avg. (SD)	3.88 (0.87)	3.81 (0.93)	3.97 (0.82)	3.9 (0.83)

Note:
Summary statistics for analysis sample.

© 2020 Solnick RE et al. *JAMA Network Open.*

Table e2: Background Characteristics by Treatment Group in Study 2

	Treatment Group			
	Simulated White Male Physician	Simulated White Female Physician	Simulated Black Female Physician	Simulated Black Male Physician
Median Age (Min, Max)	45 (18,82)	47 (18,86)	44 (18,86)	43 (18,80)
Female	218 (50%)	194 (50.39%)	179 (46.61%)	203 (51.92%)
College Educated	185 (42.43%)	156 (40.52%)	183 (47.66%)	173 (44.25%)
HHI Below Median	315 (75.36%)	268 (71.85%)	257 (68.17%)	279 (74.4%)
**Race/Ethnicity:**				
White (Non-Hispanic)	295 (67.66%)	288 (74.81%)	276 (71.88%)	274 (70.08%)
Black	57 (13.07%)	41 (10.65%)	37 (9.64%)	41 (10.49%)
Hispanic	39 (8.94%)	24 (6.23%)	41 (10.68%)	38 (9.72%)
Other	45 (10.32%)	32 (8.31%)	30 (7.81%)	38 (9.72%)
**Insurance status:**				
Medicaid	103 (23.62%)	80 (20.78%)	99 (25.78%)	88 (22.51%)
Medicare	59 (13.53%)	58 (15.06%)	62 (16.15%)	62 (15.86%)
Uninsured	51 (11.7%)	49 (12.73%)	40 (10.42%)	51 (13.04%)
**Healthcare experience:**				
Unpaid Medical Bills	99 (22.71%)	84 (21.82%)	72 (18.75%)	96 (24.55%)
1+ ED visit last 6 MOs.	108 (24.77%)	94 (24.42%)	89 (23.18%)	78 (19.95%)
**Subjective assessments:**				
Mental Health Avg. (SD)	3.5 (1.09)	3.6 (1.1)	3.57 (1.13)	3.5 (1.14)
Overall Health Avg. (SD)	3.4 (0.98)	3.44 (0.95)	3.45 (0.97)	3.36 (1.02)
Trust in Docs Avg. (SD)	3.88 (0.74)	3.9 (0.79)	3.92 (0.7)	3.8 (0.75)

Note:
Summary statistics for analysis sample.

Table e3: Randomization Inference (RI) for covariate balance

Study	Observed F-Statistic	0.025th Quantile	0.975th Quantile	RI P-value
Study 1	0.53	0.37	1.92	0.90
Study 2	1.35	0.37	1.98	0.18

Note:
Quantiles of null distribution and RI P-values from 10,000 permutations of the experimental design.

© 2020 Solnick RE et al. *JAMA Network Open.*

Table e4: Average Patient Evalution Scores on Composite Index of Primary Outcomes

	Combined (N = 3215)	Study 1 (N = 1619)	Study 2 (N = 1596)
Simulated White Male Physician	66.13 [64.76,67.51]	68.30 [66.46,70.14]	63.66 [61.63,65.70]
Simulated Black Male Physician	66.96 [65.55,68.36]	70.48 [68.51,72.44]	63.57 [61.61,65.53]
Simulated Black Female Physician	67.36 [66.03,68.69]	70.20 [68.33,72.08]	64.24 [62.40,66.08]
Simulated White Female Physician	66.50 [65.19,67.82]	71.28 [69.51,73.06]	62.29 [60.46,64.12]

Note:
Means and 95% CIs displayed for each group.

pdf 2

Table e5: Estimated Treatment Effects on Primary Outcomes in Combined Sample

	Treatment		
	Simulated White Female Physician	Simulated Black Female Physician	Simulated Black Male Physician
Composite Index	0.03 [-0.07,0.13]	0.05 [-0.05,0.15]	0.06 [-0.04,0.16]
Patient Confidence	0.02 [-0.08,0.12]	0.05 [-0.05,0.15]	0.06 [-0.04,0.16]
Patient Satisfaction	0.03 [-0.07,0.13]	0.06 [-0.04,0.16]	0.06 [-0.04,0.16]
Likelihood to Recommend	0.04 [-0.06,0.14]	0.07 [-0.03,0.17]	0.08 [-0.02,0.18]
Believes Symptom Checker	0.03 [-0.07,0.13]	0.05 [-0.05,0.15]	0.03 [-0.07,0.13]
Requests More Tests	0.03 [-0.07,0.13]	-0.04 [-0.14,0.06]	0.02 [-0.08,0.12]

Note:
Point estimates with 95% CIs. Estimates are presented as standardized effect sizes using Glass's Delta.
* denotes significance at 0.05 after Benjamini & Hochberg (1995) adjustment for multiple comparisons.

© 2020 Solnick RE et al. *JAMA Network Open.*

Table e6: Estimated Treatment Effects on Primary Outcomes in Study 1

	Treatment		
	Simulated Black Male Physician	Simulated Black Female Physician	Simulated White Female Physician
Patient Confidence	0.08 [-0.05,0.22]	0.09 [-0.04,0.22]	0.09 [-0.04,0.22]
Patient Satisfaction	0.06 [-0.07,0.19]	0.08 [-0.04,0.21]	0.10 [-0.02,0.22]
Likelihood to Recommend	0.13 [-0.01,0.27]	0.10 [-0.04,0.23]	0.10 [-0.04,0.23]
Believes Symptom Checker	0.01 [-0.12,0.14]	0.06 [-0.07,0.18]	0.10 [-0.03,0.22]
Requests More Tests	0.04 [-0.09,0.17]	-0.00 [-0.13,0.13]	0.03 [-0.11,0.16]

Note:
Point estimates with 95% CIs. Estimates are presented as standardized effect sizes using Glass's Delta.
* denotes significance at 0.05 after Benjamini & Hochberg (1995) adjustment for multiple comparisons.

Table e7: Estimated Treatment Effects on Primary Outcomes in Study 2

	Treatment		
	Simulated Black Male Physician	Simulated Black Female Physician	Simulated White Female Physician
Patient Confidence	0.03 [-0.11,0.16]	0.02 [-0.11,0.15]	-0.05 [-0.18,0.07]
Patient Satisfaction	0.04 [-0.09,0.18]	0.04 [-0.09,0.16]	-0.05 [-0.18,0.08]
Likelihood to Recommend	0.02 [-0.12,0.15]	0.05 [-0.08,0.18]	-0.02 [-0.15,0.11]
Believes Symptom Checker	0.04 [-0.11,0.18]	0.04 [-0.10,0.18]	-0.05 [-0.19,0.09]
Requests More Tests	-0.02 [-0.16,0.12]	-0.06 [-0.20,0.07]	0.00 [-0.13,0.14]

Note:
Point estimates with 95% CIs. Estimates are presented as standardized effect sizes using Glass's Delta.
* denotes significance at 0.05 after Benjamini & Hochberg (1995) adjustment for multiple comparisons.

pdf 2

© 2020 Solnick RE et al. *JAMA Network Open.*

Table e8: Summary Statistics for BART Estimated Treatment Effects on Composite Index (0-100)

	Treatment	Prop. > 0	Mean	SD	0.025th Quantile	0.975th Quantile
Study 1	Simulated White Female Physician	0.98	2.22	1.11	0.09	4.32
Study 2	Simulated White Female Physician	0.10	-1.28	1.28	-4.30	0.45
Study 1	Simulated Black Female Physician	0.82	1.62	1.67	-1.77	4.45
Study 2	Simulated Black Female Physician	0.60	0.32	1.18	-1.78	2.68
Study 1	Simulated Black Male Physician	0.93	2.07	1.33	-0.33	4.21
Study 2	Simulated Black Male Physician	0.56	0.57	1.81	-2.35	4.61

Note:
Summary statistics of BART estimated treatment effects for each subject.

Table e9: Estimated Treatment Effects on Secondary Outcomes in Study 1

	Treatment		
	Simulated Black Male Physician	Simulated Black Female Physician	Simulated White Female Physician
Perceived Warmth	0.33 [0.20,0.45]*	0.15 [0.00,0.30]	-0.02 [-0.16,0.12]
Perceived Competence	0.14 [0.02,0.27]	0.05 [-0.09,0.19]	0.10 [-0.03,0.24]
Fairness of Cost	0.05 [-0.08,0.18]	0.05 [-0.08,0.18]	0.10 [-0.04,0.23]

Note:
Point estimates with 95% CIs. Estimates are presented as standardized effect sizes using Glass's Delta.
* denotes significance at 0.05 after Benjamini & Hochberg (1995) adjustment for multiple comparisons.

© 2020 Solnick RE et al. *JAMA Network Open.*

Table e10: Estimated Treatment Effects on Secondary Outcomes in Study 2

	Treatment		
	Simulated Black Male Physician	Simulated Black Female Physician	Simulated White Female Physician
Perceived Warmth	0.09 [-0.04,0.22]	0.08 [-0.06,0.21]	-0.02 [-0.15,0.11]
Perceived Competence	0.06 [-0.08,0.19]	0.06 [-0.07,0.20]	-0.03 [-0.15,0.10]
Complain for Error	-0.07 [-0.22,0.07]	-0.01 [-0.15,0.13]	0.02 [-0.12,0.15]
Sue for Error	-0.06 [-0.20,0.08]	-0.02 [-0.17,0.12]	-0.01 [-0.15,0.12]

Note:
Point estimates with 95% CIs. Estimates are presented as standardized effect sizes using Glass's Delta.
* denotes significance at 0.05 after Benjamini & Hochberg (1995) adjustment for multiple comparisons.

© 2020 Solnick RE et al. *JAMA Network Open.*

10 References

1. W. Lin, Agnostic notes on regression adjustments to experimental data: Reexamining freedman's critique. *The Annals of Applied Statistics.* **7**, 295–318 (2013).

2. D. M. Oppenheimer, T. Meyvis, N. Davidenko, Instructional manipulation checks: Detecting satisficing to increase statistical power. *Journal of Experimental Social Psychology.* **45**, 867–872 (2009).

3. A. W. Meade, S. B. Craig, Identifying careless responses in survey data. *Psychological methods.* **17**, 437 (2012).

4. A. J. Berinsky, G. A. Huber, G. S. Lenz, Evaluating online labor markets for experimental research: Amazon.com's mechanical turk. *Political Analysis.* **20**, 351–368 (2012).

5. D. J. Hauser, N. Schwarz, Attentive turkers: MTurk participants perform better on online attention checks than do subject pool participants. *Behavior research methods.* **48**, 400–407 (2016).

6. P. M. Aronow, J. Baron, L. Pinson, A note on dropping experimental subjects who fail a manipulation check. *Political Analysis*, 1–18 (2018).

7. J. A. Hall, J. T. Irish, D. L. Roter, C. M. Ehrlich, L. H. Miller, Satisfaction, gender, and communication in medical visits. *Medical care* (1994).

8. A. S. Gerber, D. P. Green, *Field experiments: Design, analysis, and interpretation* (WW Norton, 2012).

9. L. Huddy, S. Feldman, On assessing the political effects of racial prejudice. *Annual Review of Political Science.* **12**, 423–447 (2009).

10. K. Peyton, G. A. Huber, Do survey measures of racial prejudice predict racial discrimination? Experimental evidence on anti-black discrimination. *SocArXiv* (2018) (available at https://osf.io/preprints/socarxiv/qwusz/).

11. P. Glick, S. T. Fiske, The ambivalent sexism inventory: Differentiating hostile and benevolent sexism. *Journal of personality and social psychology.* **70**, 491 (1996).

12. J. R. Kunst, R. Fischer, J. Sidanius, L. Thomsen, Preferences for group dominance track and mediate the effects of macro-level social inequality and violence across societies. *Proceedings of the National Academy of Sciences.* **114**, 5407–5412 (2017).

13. S. T. Fiske, A. J. Cuddy, P. Glick, J. Xu, A model of (often mixed) stereotype content: Competence and warmth respectively follow from perceived status and competition. *Journal of personality and social psychology.* **82**, 878 (2002).

14. G. T. Kraft-Todd *et al.*, Empathic nonverbal behavior increases ratings of both warmth and competence in a medical context. *PloS one.* **12**, e0177758 (2017).

15. S. T. Fiske, C. Dupree, Gaining trust as well as respect in communicating to motivated audiences about science topics. *Proceedings of the National Academy of Sciences.* **111**, 13593–13597 (2014).

 © 2020 Solnick RE et al. *JAMA Network Open.*
