# Supplementary material for: Effect of Physician Gender and Race on Simulated Patients’ Ratings and Confidence in Their Physicians: A Randomized Trial
Source: JAMA Netw Open. 2020 Feb 21;3(2):e1920511. doi: 10.1001/jamanetworkopen.2019.20511 (PMC7043197; doi:10.1001/jamanetworkopen.2019.20511)
Supplement: Supplement 3. — Data Sharing Statement [file jamanetwopen-3-e1920511-s003.pdf]

Data Sharing Statement

Solnick. Effect of Physician Gender and Race on Simulated Patients' Ratings and Confidence in Their Physicians: A Randomized Trial. *JAMA Netw Open*. Published February 21, 2020. 10.1001/jamanetworkopen.2019.20511

Data available: Yes

Data types: Deidentified participant data

How to access data: Data will be available at Harvard Dataverse. https://doi.org/10.7910/DVN/GUAFU0

When available: With publication

Document types: Statistical/analytic code

How to access documents: https://doi.org/10.7910/DVN/GUAFU0

When available: With publication

Who can access the data: Anyone with access to the internet.

Types of analyses: Replication of all statistical analyses reported in the paper and eMethods

Mechanisms of data availability: Not applicable. No restrictions will be placed on data access.
